# Nurse Managers' Awareness of and Competence in Palliative Care in Municipal Care Settings: A Systematic Mixed Studies Review

**DOI:** 10.1111/nhs.70352

**Published:** 2026-05-12

**Authors:** Hongxuan Xu, Marie‐Louise Möllerberg, Karin Dalhammar, Mariette Bengtsson, Katarina Sjögren Forss

**Affiliations:** ^1^ Department of Care Sciences Faculty of Health and Society, Malmö University Malmö Sweden

**Keywords:** awareness, competence, municipal care, nurse manager, palliative care, systematic review

## Abstract

Nurse managers' role in providing high‐quality palliative care is crucial for addressing the increasing demand in municipal care. This study aims to compile existing evidence on nurse managers' awareness of and competence in palliative care in municipal care settings. The systematic review followed the Preferred Reporting Items for Systematic Reviews and Meta‐Analyses recommendations and was registered in PROSPERO (CRD42024559183). In total, 6289 articles were initially retrieved, and 489 additional articles were identified in the updated searches. A narrative synthesis was conducted. Eight studies met the inclusion criteria and three themes emerged from the narrative synthesis: (1) Conceptual grasp of palliative care, (2) Recognition of gaps and needs, and (3) Competence in fulfilling complex responsibilities. Nurse managers' understanding of palliative care along with identified gaps and priorities shaped their competence in managing complex responsibilities related to palliative care, where confidence influenced how they performed the responsibilities. The results pointed to a knowledge gap in the explored area. Future research is needed to explore nurse managers' perspectives on how they understand and perform their leadership role in relation to palliative care.

## Key Points

- The results showed a knowledge gap regarding how nurse managers perceive and influence palliative care practices in municipal care settings.
- Nurse managers' conceptual understanding and interpretation of palliative care shaped their focus and priorities in relation to palliative care practices.
- Nurse managers' competence in palliative care was demonstrated in performing their diverse responsibilities and was closely tied to their confidence in leading complex care processes.

**Abbreviations**
NM: nurse manager
PC: palliative care
RN: registered nurse

## 1 Background

As medical advances and life expectancy increase among an aging population, the need and demand for palliative care (PC) have grown substantially on a global scale (World Health Organisation 2020; Albers et al. 2015). Ensuring the standard and quality of PC through PC leadership is imperative. Nurse managers (NMs) play a pivotal role in overseeing the delivery and quality of care provided by registered nurses (RNs) (Andersson et al. 2017; Nilsson et al. 2009), fostering evidence‐based practice within care organizations (Kvist et al. 2014; Sandström et al. 2011), and enabling the delivery of high‐quality, compassionate, person‐centred care (McSherry et al. 2012; Vesterinen et al. 2013). Thus, NMs' competence is not only crucial for ensuring quality of care but also a key driver in advancing the professional growth of RNs in the field and maintaining a well‐functioning care system (Amankwah et al. 2022; World Health Organisation 2023).

Palliative care is based on an integrated and holistic approach focusing on relieving symptoms, optimizing the quality of life, and addressing the complex needs of patients and their relatives across all stages of life‐threatening illnesses and for patients of all ages (Lavoie et al. 2013; Randall and Downie 2006). Person‐centred PC is essential to prioritise the individual needs and preferences of patients and their relatives in decision‐making, planning, and resultant health and social care (Lavoie et al. 2013; Österlind and Henoch 2021). Unlike specialist PC, which is delivered by a multidisciplinary team with advanced PC knowledge and expertise, general PC is often provided by healthcare professionals from nonspecialist PC teams as an integral part of standard clinical practice and can be provided in most forms of care (Radbruch and Sa 2010; Payne et al. 2022).

The aging population has led to a growing number of older persons with complex care needs requiring PC. The World Health Organization (World Health Organisation 2023) has emphasized the importance of integrating PC into primary care as well as community and home care. Municipal care typically refers to health and social services provided by local governments (i.e., municipalities) to every resident in need, for instance, in the form of nursing homes, rehabilitation, and home care services. A competent nursing workforce is vital to achieving the integration of PC, as it constitutes the largest professional group in general PC provision (Hökkä et al. 2020; Martins Pereira et al. 2021). Nursing competence in PC encompasses multiple aspects, including the ability to collaborate with patients, families, and the care team; communication and cultural sensitivity; clinical skills; psychosocial and spiritual competency; ethical and legal competency; and competencies related to professional roles and leadership (Hökkä et al. 2021). Registered nurses in municipal care are responsible for the healthcare of older persons in both residential facilities and home care settings (Sneltvedt and Bondas 2016). They often care for patients with complex PC needs associated with advanced ages, requiring broader perspectives, approaches, and considerations in care (Hallberg 2006). Research demonstrates a wide disparity in quality indicators and structural characteristics in care services operated by highly autonomous municipalities across countries (Rostad et al. 2023), resulting in variations in the model of care delivery and in the allocation of care resources (Maetens et al. 2017; Burrell et al. 2023). Thus, the role of NMs in municipal care generally involves a diverse range of organizational, administrative, and clinical functions and responsibilities, all aimed at providing high‐quality care and supporting RNs on the unit by building, developing, and leading the teams (Dwyer 2011; Solbakken et al. 2019).

Municipal care settings tend to be characterized by a vague distribution of responsibilities and unique care environments (Kröger and Puthenparambil 2018). Registered nurses in municipal care often have a significant need for more profound knowledge of PC and support from their managers in order to deliver high‐quality PC (Kröger and Puthenparambil 2018; Asante et al. 2021). Studies indicate that RNs working in municipal care have frequently reported moral distress and dissatisfaction with their work situation, primarily due to limited resources in terms of knowledge, education, and equipment, lack of support from their colleagues and managers, as well as limited influence over their work conditions, hindering their ability to provide high‐quality care (Munkeby et al. 2023; Josefsson et al. 2008). Maintaining and enhancing the quality of PC through promoting the PC competence of RNs is one of the key responsibilities of NMs, as they need to ensure that their units are staffed with sufficiently skilled RNs and that these nurses work under optimal working conditions (Arvidsson and Fridlund 2005; Nurmeksela et al. 2021). However, this is attainable only if NMs themselves have adequate awareness of and robust competence in PC (World Health Organisation 2023; Batt et al. 2020). It is consequently necessary and important for NMs to be highly aware of and possess PC competence grounded in clinical practice to enable them to facilitate their effective professional practices, including developing advanced management and leadership strategies, and to ensure high‐quality care and optimal healthcare workforce performance (Batt et al. 2020; González‐García et al. 2021).

The concept of competence in nursing and healthcare has been defined variously but is widely viewed through a common lens, incorporating several interconnected dimensions, such as knowledge, skills, abilities, values, beliefs, attributes, and behaviors, essential for effectively performing specific roles and managing various tasks or situations (Cowan et al. 2007; Becker 2010). When engaging in such roles, individuals may get a sense of whether they are competent and capable of fulfilling particular expectations related to their professional responsibilities (Bandura 1982; Van Dyk et al. 2016). This sense, referred to as self‐efficacy or confidence, is meanwhile shaped through social and environmental influences (Bandura 1982). Published research widely recognizes the role of confidence in both the development of competence and its demonstration in evaluative contexts (Cohen et al. 2013). Becker (2007) further emphasizes the importance of adopting a holistic perspective on what constitutes competence within a professional context, in order to enhance our understanding of its meaning and application. Considering NMs' multifaceted roles with varying responsibilities in municipal care, their competence in PC should enable them to effectively meet the complex and increasing PC needs while maintaining a supportive care environment (Bandura 1982; Van Dyk et al. 2016). Equally important as competence is awareness, as it represents the knowledge or perception of a fact while reflecting the recognition of one's own knowledge gaps and deficiencies, often serving as the first essential step in developing competence (Adams 2011).

The shift in the balance of care from the hospital to the community, in alignment with policies and guidelines for integrating PC into community‐based care, has led to evolving expectations regarding the roles, responsibilities, and competence required of NMs, pointing to the unique challenges that NMs face in municipal care (World Health Organisation 2020; Haycock‐Stuart and Kean 2012; Søreide et al. 2019). A recent review suggests that the unpreparedness of healthcare professionals to deal with the growing need for PC may stem from deficiencies in managers' competence (Silva et al. 2021). The competence gap in PC has been identified among the managers of assistant nurses in nursing homes, who reported that limited knowledge of PC constrained their ability to fulfill leadership responsibilities in this area (Håkanson et al. 2014). Such concerns highlight the importance of all workforces in municipal care having basic PC competence, especially RNs who represent the largest professional group involved in general PC. Despite the shifting demands in municipal care, there remains a large gap in the comprehensive understanding of the PC awareness and competence that NMs possess. Additionally, to the best of the authors' knowledge, there is no systematic review outlining the knowledge in this area. Therefore, this study aims to compile existing evidence on NMs' awareness of and competence in palliative care in municipal care settings.

## 2 Methods

A systematic mixed studies review was undertaken to synthesize and analyze current evidence on NMs' awareness of and competence in PC. The review followed the Preferred Reporting Items for Systematic Reviews and Meta‐Analyses (PRISMA) reporting guideline (Liberati et al. 2009; Page et al. 2021) (see File S1). The review protocol is registered in PROSPERO (CRD42024559183).

### 2.1 Eligibility Criteria

Study characteristics were identified by the Sample, Phenomenon of Interest, Design, Evaluation, Research type (SPIDER) tool to design review questions and as a means to develop and standardize the search strategies for qualitative, quantitative, and mixed‐method studies (Cooke et al. 2012). Nurse managers were defined as managers or leaders of RNs working in municipal care settings. Awareness of PC was conceptualized as how individuals consciously understand and perceive PC and its principles as well as reflect on their own knowledge and context. Competence in PC referred to the ability to perform roles and manage tasks or situations in relation to PC, encompassing knowledge, skills, abilities, values, beliefs, attributes, and behaviors essential for professional practice. The inclusion and exclusion criteria based on SPIDER are presented in Table 1.

**TABLE 1:** Inclusion and exclusion criteria based on the SPIDER tool.

SPIDER tool		Inclusion criteria	Exclusion criteria
Sample	Nurse managers working in municipal care settings.	Studies involving the managers of RNs working in municipal care settings (e.g., community care, home care, nursing homes or other facilities carried out by municipalities).	Studies including only organization managers; studies including managers in hospitals or care settings outside the municipality, or managers of professionals other than nurses.
Phenomenon of interest	Nurse managers' awareness of PC and competence in PC (knowledge, skills, attitudes, and behaviors)	Studies that focus on how NMs manage and lead PC in municipal care; Studies that focus on how NMs manage and lead municipal care while also mentioning PC; studies that focus on the PC competence required or exhibited in municipal care.	Studies conducted outside of municipal care settings (e.g., in regions or private sectors), providing no relevant insights into competence applicable to municipal care; studies focusing on PC broadly but not considering the competence of NMs; studies focusing on nursing competence in PC but not targeting NMs; Studies focusing only on specialist PC; studies focusing on end‐of‐life issues or advanced planning generally; studies focusing on the COVID‐19 pandemic or its related response strategies.
Design	Qualitative, quantitative, mixed‐methods, and comparative studies.	Qualitative, quantitative, mixed‐methods, and comparative approaches or methodologies.	Non‐empirical studies (e.g., reviews, meta‐analyses, discussion papers, commentaries, methodological or theoretical studies); intervention studies.
Evaluation	Measurements, assessments, descriptions, illustration, or elaboration of general PC awareness and competence.	Studies that address the awareness of PC, including the recognition, consciousness, understandings, or perceptions of PC; studies that address the competence related to PC, including the aspects of knowledge, skills, attitudes, and behaviors.	Studies focusing only on specialist PC competence, organizational competence development, or intervention outcomes/effects on PC competence.
Research type	Peer‐reviewed. Empirical studies.	Peer‐reviewed empirical studies; studies published in English; full‐text available.	Unpublished studies or reports; gray literature.

### 2.2 Search Strategy

The search was conducted in PubMed, CINAHL Complete, PsycInfo, Scopus, and ABI/INFORM Global. A test search was performed prior to the systematic search strategy to identify relevant subject terms and free text terms (Liberati et al. 2009; Page et al. 2021). Based on the discussion in the research group and with the assistance of two librarians from Malmö University, a systematic search strategy was developed (see File S2). In each of the databases, a building block search was performed, where the Medical Subject Headings were combined with title/abstract search terms by Boolean operators, that is OR and AND, to ensure the specificity within the search strategy (Shaw et al. 2004). All databases were searched using the same title/abstract search terms taking into account word forms and synonyms. However, some search terms were slightly adjusted to suit the specific characteristics of each database. Although peer‐review filters were applied during searches in three databases (i.e., CINAHL Complete, PsycInfo, and ABI/INFORM Global), all publications were further assessed for adherence to peer‐reviewed standards during the study selection process. The last search in all databases was performed on 2024‐10‐24. In total, 6289 articles were retrieved from the databases. Additional searches, including a citation pearl search in Web of Science and a manual search in six reviews, conducted on 2025‐01‐14, did not identify any additional relevant articles. An updated search was performed on 2026‐01‐09, where a total of 489 new articles were found.

### 2.3 Study Selection

Covidence software was used for the screening and selection process (Covidence Systematic Review Software 2025). Of the 6289 articles retrieved, 2617 were duplicates, 2533 of which were identified by Covidence and 84 identified manually during the screening process. The updated search resulted in the inclusion of one additional article for quality appraisal.

**FIGURE 1:**
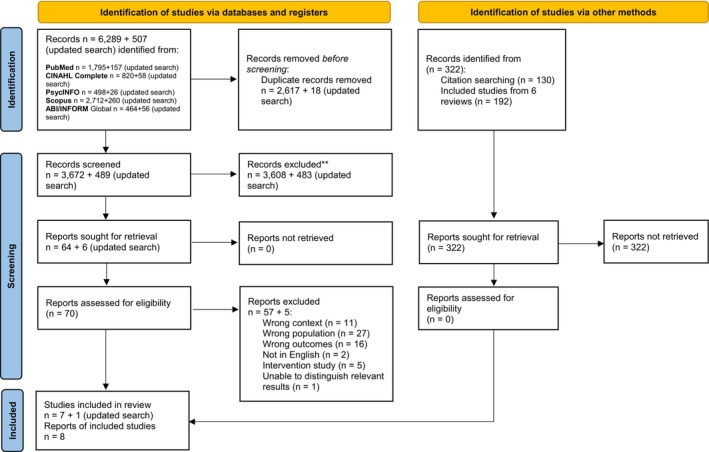
PRISMA flow diagram. *Consider, if feasible to do so, reporting the number of records identified from each database or register searched (rather than the total number across all databases/registers). **If automation tools were used, indicate how many records were excluded by a human and how many were excluded by automation tools. *Source:* Page MJ, et al. BMJ 2021;372: n71. Doi:10.1136/bmj.n71 . This work is licensed under CC BY 4.0. To view a copy of this license, visit https://creativecommons.org/licenses/by/4.0/.

The entire study selection, including the updated search, was undertaken collaboratively by at least two authors. They first screened the titles and abstracts of the articles individually, and then the studies that met the inclusion criteria in screening proceeded to full text review. In case of disagreement during the screening, there was a discussion between the two authors until consensus was reached. Both authors agreed to read the full texts of 64 articles. They then reported the screening process in a meeting with the other authors (*n* = 3), reflecting on their roles and experiences in the screening process. The full texts were distributed among the authors, ensuring that each article was reviewed by at least two authors. Studies excluded during this process were noted with reasons. In case of disagreement regarding either inclusion or differing exclusion reasons during the full‐text review, the two authors responsible for the article in question discussed the rationale for their votes. If uncertainty remained between them, the discussion was extended to the whole group until consensus was reached. The entire study selection process is presented in a PRISMA flow diagram, see Figure 1.

### 2.4 Quality Appraisal

To ensure the rigor and reliability of the included studies, a systematic quality appraisal was conducted (Liberati et al. 2009; Page et al. 2021). The appraisal tools were selected to accommodate the diverse study designs included in this review. Four authors independently assessed all included articles using the respective appraisal tools, demonstrating a high degree of consistency in their evaluations of study quality. Consensus on the quality of each study was easily reached through discussion. The quality of each study was presented in Table 2.

**TABLE 2:** Quality appraisal.

JBI checklist for qualitative studies	Bükki et al. (2016)	Katz et al. (2000)	Arjama et al. (2025)
1. Is there congruity between the stated philosophical perspective and the research methodology?	Unclear	Unclear	Unclear
2. Is there congruity between the research methodology and the research question or objectives?	No	Yes	Yes
3. Is there congruity between the research methodology and the methods used to collect data?	Yes	Yes	Yes
4. Is there congruity between the research methodology and the representation and analysis of data?	Yes	No	Yes
5. Is there congruity between the research methodology and the interpretation of results?	Yes	No	Yes
6. Is there a statement locating the researcher culturally or theoretically?	Unclear	No	Yes
7. Is the influence of the researcher on the research, and vice‐versa, addressed?	Unclear	No	Yes
8. Are participants, and their voices, adequately represented?	Yes	Yes	Yes
9. Is the research ethical according to current criteria or, for recent studies, and is there evidence of ethical approval by an appropriate body?	Yes	No	Yes
10. Do the conclusions drawn in the research report flow from the analysis, or interpretation, of the data?	Yes	Yes	Yes
Overall quality	6/10 60% moderate	4/10 40% low	9/10 90% high

Appraisal tool for cross‐sectional studies (axis)		De Bellis and Parker (1998)	Eriksson et al. (2015)	Mitchell et al. (2016)
Questions	Question grouping*			
**Introduction**				
1. Were the aims/objectives of the study clear?	Reporting quality	Yes	Yes	Yes
**Methods**				
2. Was the study design appropriate for the stated aim(s)?	Study design	Yes	Yes	Yes
3. Was the sample size justified?	Study design	No	No	Yes
4. Was the target/reference population clearly defined? (Is it clear who the research was about?)	Reporting quality	Yes	Yes	Yes
5. Was the sample frame taken from an appropriate population base so that it closely represented the target/reference population under investigation?	Study design	Yes	Yes	No
6. Was the selection process likely to select subjects/participants that were representative of the target/reference population under investigation?	Risk of bias	Yes	Yes	No
7. Were measures undertaken to address and categorize non‐responders?	Risk of bias	No	Yes	No
8. Were the risk factor and outcome variables measured appropriate to the aims of the study?	Study design	Yes	Yes	Yes
9. Were the risk factor and outcome variables measured correctly using instruments/measurements that had been trialed, piloted or published previously?	Risk of bias	Don't know	Yes	Don't know
10. Is it clear what was used to determined statistical significance and/or precision estimates? (e.g., *p* values, confidence intervals)	Reporting quality	No	Yes	No
11. Were the methods (including statistical methods) sufficiently described to enable them to be repeated?	Reporting quality	Yes	Yes	Yes
**Results**				
12. Were the basic data adequately described?	Reporting quality	No	Yes	No
13. Does the response rate raise concerns about non‐response bias?**	Risk of bias	Yes	No	No
14. If appropriate, was information about non‐responders described?	Risk of bias	No	Yes	No
15. Were the results internally consistent?	Risk of bias	No	Yes	Yes
16. Were the results presented for all the analyses described in the methods?	Reporting quality	No	Yes	Yes
**Discussion**				
17. Were the authors' discussions and conclusions justified by the results?	Study design	Yes	Yes	Yes
18. Were the limitations of the study discussed?	Reporting quality	No	Yes	Yes
**Other**				
19. Were there any funding sources or conflicts of interest that may affect the authors' interpretation of the results?**	Study design	Don't know (not stated)	Don't know	No
20. Was ethical approval or consent of participants attained?	Study design	Yes	Yes	Yes
Overall quality		9/20 45% low	18/20 90% high	13/20 65% moderate

Mixed methods appraisal tool (MMAT)		Froggatt and Payne (2006)	Tunnard et al. (2024)
Screening questions	S1. Are there clear research questions?	Yes	Yes
	S2. Do the collected data allow to address the research questions?	Yes	Yes
Qualitative	1.1. Is the qualitative approach appropriate to answer the research question?	Yes	Yes
	1.2. Are the qualitative data collection methods adequate to address the research question?	Yes	Yes
	1.3. Are the findings adequately derived from the data?	Yes	Yes
	1.4. Is the interpretation of results sufficiently substantiated by data?	Yes	Yes
	1.5. Is there coherence between qualitative data sources, collection, analysis and interpretation?	Yes	Yes
Quantitative descriptive	4.1. Is the sampling strategy relevant to address the research question?	Yes	No
	4.2. Is the sample representative of the target population?	Yes	Yes
	4.3. Are the measurements appropriate?	No	Yes
	4.4. Is the risk of nonresponse bias low?	No	Can't tell
	4.5. Is the statistical analysis appropriate to answer the research question?	Yes	Yes
Mixed methods	5.1. Is there an adequate rationale for using a mixed methods design to address the research question?	No	No
	5.2. Are the different components of the study effectively integrated to answer the research question?	No	Yes
	5.3. Are the outputs of the integration of qualitative and quantitative components adequately interpreted?	Yes	Yes
	5.4. Are divergences and inconsistencies between quantitative and qualitative results adequately addressed?	Can't tell	Yes
	5.5. Do the different components of the study adhere to the quality criteria of each tradition of the methods involved?	Yes	Yes
Overall quality		5 + 3 + 2 40% low	5 + 3 + 4 60% moderate

* Question grouping: Reporting quality (Questions 1, 4, 10, 11, 12, 16, 18); Study design (Questions 2, 3, 5, 8, 17, 19, 20); Risk of bias (Questions 6, 7, 9, 13, 14, 15).

** Question is reverse scored (i.e., ‘no’ means positive; ‘yes’ means negative).

The Appraisal tool for Cross‐Sectional Studies (AXIS) was used to appraise three studies with a quantitative design (Downes et al. 2016). AXIS contains 20 questions addressing study design (seven questions), reporting quality (seven questions), and risk of bias (six questions) in cross‐sectional studies (Ma et al. 2020). The questions were answered with “yes”, “no” or “don't know”. AXIS does not provide a numerical summary score of overall quality, due to a concern about the variability and inconsistency of summary scores across scales (Greenland and O'Rourke 2001; Jüni et al. 1999; Sanderson et al. 2007). Instead, AXIS allows for the evaluation of each aspect of a study to form a judgment on overall quality (Downes et al. 2016). Each criterion was assessed as either met (a “yes” response) or not met (a “no” or “don't know” response) (Rovito et al. 2021) (Henderson et al. 2019; Wong et al. 2018), except for questions 13 and 19, which were assessed using reverse scoring (Downes et al. 2016; Wong et al. 2018). This review aligns with previous systematic reviews, in which a predetermined percentage score was used to assess study quality, defining high‐quality articles as those meeting at least 70% of the criteria (i.e., 14 or more questions) (Rovito et al. 2021). Articles meeting between 50% and 70% of the criteria were classified as moderate quality, whereas those meeting less than 50% were considered low quality (Rovito et al. 2021).

The Joanna Briggs Institute (JBI) Critical Appraisal Checklist for Qualitative Research was used to appraise the quality of three qualitative studies, including one from the updated search. The checklist contains 10 questions assessing the study's congruity, methodology, the researcher's role, rigor of data interpretation, and coherence of the conclusions (Lockwood et al. 2015). The questions were answered with “yes”, “no”, “unclear” or “not applicable”. JBI suggests that the overall quality assessment of each study can be conducted based on either meeting a predetermined proportion of all criteria or ensuring that specific essential criteria are satisfied (Aromataris et al. 2024). As JBI is designed to cumulatively assess individual characteristics of the studies, there are no established rules for determining the quality of each study. Aligning with previous systematic reviews (Whittaker et al. 2022; Franco et al. 2020), a percentage score was used to classify study quality based on all applicable questions, excluding those marked as “not applicable”. In this review, studies scoring 70% or more were classified as “high quality”, those scoring between 50% and 70% were categorized as “moderate quality”, and studies scoring below 50% were considered as “low quality”.

The Mixed Methods Appraisal Tool (MMAT) was used to appraise the quality of three mixed‐methods studies (Hong et al. 2018). The MMAT consists of screening questions (2 questions), qualitative components (5 questions), design‐specific quantitative components (5 questions), and mixed‐methods components (5 questions). The MMAT suggests that, in a mixed‐methods study design, the overall quality of the study cannot exceed the quality of its weakest component, meaning the overall quality score is determined by the lowest score among the qualitative, quantitative, and mixed‐methods components (Hong et al. 2018). A percentage score of each study was thus generated based on the component with the lowest score. The MMAT does not define cut‐off values to categorize studies as low, moderate, or high quality. Instead, it emphasizes the importance of transparently describing how the appraisal results were interpreted and applied in the review. Consistent with previous systematic reviews (Scott et al. 2019; Morrison et al. 2022), three categories were used to define study quality: high quality (80% or higher) if four or more questions met the criteria in the component with the lowest score, moderate quality (between 40% and 80%) if two or three questions met the criteria in the component with the lowest score, and low quality (40% or below) if fewer than two questions met the criteria in the component with the lowest score. At this stage, one mixed‐methods study was excluded due to the unavailability of clearly distinguishable relevant data, by consensus of all authors. This excluded study was noted in Figure 1 and the reason for exclusion is “unable to distinguish relevant results”.

### 2.5 Data Extraction and Synthesis

Narrative synthesis was employed to analyze and integrate findings from the included studies that incorporated both qualitative and quantitative data (Popay et al. 2006; Pope et al. 2007). Popay et al. described narrative synthesis as an iterative and flexible approach that can be adapted to the nature of a particular review. Narrative synthesis provides a systematic, transparent, and flexible approach to integrating and interpreting qualitative and quantitative findings while accounting for the heterogeneity and complexity in study natures (Popay et al. 2006; Pope et al. 2007; Chandler et al. 2019). The narrative synthesis was guided by three elements that were conducted iteratively and concurrently, namely, developing a preliminary synthesis of the findings of included studies, exploring relationships in the data, and assessing the robustness of the synthesis (Popay et al. 2006). As the review aimed to compile existing evidence broadly rather than build upon or develop a theory, the theory development element of Popay et al. narrative synthesis framework was not undertaken.

#### 2.5.1 Developing a Preliminary Synthesis of the Findings of Included Studies

A structured data extraction table was created based on the SPIDER model and Cochrane recommendations (Li and Deeks 2019). The study characteristics include authors, year of publication, study aim/research question, setting, population, method, main findings, and limitations. The extracted data were presented in Table 3 and were checked for accuracy by all authors. The findings of each study were presented in a textual description. For the qualitative data, an inductive thematic synthesis following Thomas and Harden's approach (Thomas and Harden 2008) was conducted in order to systematically organize findings and identify key themes from the extracted qualitative data before deeper comparisons occurred. The thematic synthesis started with line‐by‐line coding of the findings of all primary qualitative studies, generating initial codes to capture key concepts in the content. These codes were then organized into descriptive themes by identifying similarities and differences between codes and grouping the initial codes. Finally, the analytical themes were developed based on the descriptive themes and the aim of the review (Thomas and Harden 2008). Studies including quantitative data showed variations in their measurement tools, statistical methods, and results presentations. Therefore, the quantitative descriptive data extracted from the included studies were transformed into percentage values to show patterns, trends, and comparisons in the data. These quantitative data were subsequently classified and categorized under the corresponding qualitative themes at a descriptive level to facilitate the exploration of similarities and differences between the studies (Popay et al. 2006) (Table 4).

**TABLE 3:** Study characteristics.

Authors, year of publication, county	Study aims/research questions	Study design	Settings	Population	Data collection	Data analysis	Main findings	Quality appraisal
Arjama et al. (2025) Findland	To describe the types of ethical issue that NMs encounter in their work	Qualitative Interview study	Long‐term care settings in municipalities	Nurse managers (*n* = 23)	Focus group interviews	Inductive Content Analysis	Nurse managers needed to be aware of the care decisions and ensured that ethical high‐quality person‐centred care was provided to residents and families. They needed to ensure that family members and residents were aware of end‐of‐life care. They aimed to provide ethical leadership but require support from senior managements. Nurse managers reported that the lack of social discourse about end‐of‐life affected the resource allocation from the systems.	High quality
Bükki et al. (2016) Germany	To explore views, attitudes, and concerns among staff and to embark on a process that facilitates end‐of‐life care on an institutional level.	Qualitative study within an action research framework	Municipal nursing home	Nursing home staff (*n* = 22), including nurses, care managers, and physicians	Focus group interviews	Thematic content analysis	Managers recognized the value of palliative care and emphasized the importance of palliative care teams involvement, supporting the role of mobile palliative care teams. They were confident in their knowledge and skills in end‐of‐life care but acknowledged key barriers such as workload constraints, interprofessional collaboration challenges, and legal concerns, which at times resulted in unnecessary hospital admissions. There was wide agreement among all participants on implementing reflective debriefing groups after each resident's death as a key intervention to strengthen teamwork and support staff well‐being.	Moderate quality
De Bellis and Parker (1998) Australia	To describe the palliative care activitiy and barriers to the provision of palliative care in Australian nursing homes.	Quantitative study	Nursing homes	Directors of nursing (*n* = 147)	Questionnaires assessing palliative care activities, including transfers, death and turnover, and palliative care education; barriers to providing palliative care and palliative care support.	Descriptive statistics	Most Directors of Nursing (DONs) viewed palliative care as part of their routine work, delivered in collaboration with doctors and families, and tailored to individual residents. However, a substantial percentage (76%) of residents receiving palliative care were transferred to acute hospitals. While 69% of DONs had attended palliative care training, only 16% held formal qualifications, and most felt that the current education resources for staff were inadequate. The majority of DONs indicated that the key barriers to effective palliative care included lack of resources (71%), inadequate education (47%), and professional conflicts (18%).	Low quality
Eriksson et al. (2015) Sweden	To describe the total staff working in different care organizations in a rural community in Sweden and to explore palliative care competence. The specific objectives were to describe the educational gaps, the need for support and reflection, and whether there were differences in care organizations, professions, age, and gender.	Quantitative study (April– June 2010)	Nursing homes, home care, and group residential settings	Registered nurses (*n* = 70), assistant nurses (*n* = 916), managers (*n* = 43), and paramedics (*n* = 33)	Questionnaires assessing palliative care competence, including education and support needs	Descriptive and correlational statistics	Managers recognized the importance of palliative care but lacked palliative care education, which made it challenging to effectively guide and support their teams. Half (50%) of managers had attended intermediate palliative care workshops, and a third (33%) lacked education in palliative care. Managers acknowledged the need for improved palliative care education and the support of consulting specialists in palliative care.	High quality
Froggatt and Payne (2006) UK	The aim was to describe the provision of end‐of‐life care for older people residing in care homes from the perspective of care home managers.	Mixed‐methods design as part of the first phase of a larger action research project.	Care homes	Care home managers (*n* = 81)	Questionnaire surveys	Descriptive statistics, cross tabulations, and content analysis	Nurse managers in care homes had diverse understandings of end‐of‐life care. Some viewed it as a continuation of general care, while others associated it strictly with the final days before death, with only one manager explicitly mentioning palliative care. Structured consultations with residents about their end‐of‐life preferences were limited, with only 2.3% of residents having advance directives. Managers also highlighted limited resources, facilities, and equipment to care for end‐of‐life residents in care homes compared to other community care settings.	Low quality
Katz et al. (2000) UK	The article reports findings from a study in the UK, which investigated the case for applying the principles and practices of palliative care to caring for older people dying in residential and nursing homes.	Qualitative study in a larger mixed methods study	Nursing, dual‐registered and residential homes	Stage 2: Managers of residential, nursing, and dual‐registered homes (*n* = 100). Stage 3: Residents, staff, relatives, and external professionals (e.g., doctors, community nurses, and palliative care teams) from 12 selected homes.	Stage 2: Structured and semi‐structured interviews with managers. Stage 3: Case studies conducted in 12 homes involving participant observation and formal/informal interviews with staff, residents, relatives, and external professionals.	Qualitative analysis and statistical analysis	Most managers maintained that their role was to support staff, other residents, and relatives following the death of a resident, and felt responsible for supporting bereaved relatives. The managers identified communication as a crucial skill in providing support but noted a lack of formal training in bereavement care for staff. In addition, managers acknowledged the need for practical and emotional support for staff and surviving residents but felt unable to provide adequate bereavement care due to time constraints, limited skills, and staffing shortages.	Low quality
Mitchell et al. (2016) Ireland	The aim of this study was to provide a snapshot of the level of knowledge and understanding around palliative and end‐of‐life care from care home managers from the independent sector in Northern Ireland.	Quantitative study	Care homes	Care home managers (*n* = 56)	The Palliative Care Quiz for Nursing (PCQN), a questionnaire assessing the knowledge and understanding of palliative care.	Statistical analysis	Care home managers demonstrated their level of palliative care knowledge on the PCQN, with an average score of 12.89 out of 20 (64.45%). They had a good understanding of palliative care principles, therapeutic pain‐relieving strategies, and the emotional and psychological aspects of care. However, significant knowledge gaps were identified in the biological and physical components of care.	Moderate quality
Tunnard et al. (2024) UK	To understand the influence of care home registration type (nursing, residential or dual registered) and size on senior care leader's confidence to provide palliative and end‐of‐life care	Explanatory sequential mixed methods design	Care homes, including nursing homes, dual‐registration homes, and residential homes.	Survey: Managers (*n* = 86) including home managers (*n* = 76) and deputy managers (*n* = 10), registered nurses (*n* = 8) and others (*n* = 13) Interviews: Managers (*n* = 19) including home managers (*n* = 16) and deputy managers (*n* = 3), registered nurses (*n* = 4) and others (*n* = 4)	The Palliative Care Self Efficacy Scale assessing confidence in providing palliative and end‐of‐life care. Qualitative interviews.	Statistical analysis and framework analysis	Nursing home managers reported significantly higher confidence scores on the Palliative Care Self‐Efficacy Scale compared to residential care home managers. Managers in large care homes with on‐site registered nurses reported greater preparedness, due to structured processes and access to necessary equipment. Managers identified that confidence and preparedness in providing palliative care were influenced by training and access to external palliative care services, particularly the availability of ad‐hoc support from external providers. Partnerships with external services, such as GPs and palliative care teams, were crucial for supporting palliative care delivery, with nursing home managers benefiting from established relationships that facilitated anticipatory prescribing and symptom management. Managers emphasized the importance of structured guidance, standardized care planning, and training to enhance confidence in providing palliative care. All nursing home managers and 88% of dual‐registered home managers used formal guidance to support palliative care delivery.	Moderate quality

**TABLE 4:** Quantitative findings.

Study	Quantitative texual descriptions of data		Transformed percentage values	Integration in the themes
De Bellis and Parker (1998)	13% of DONs would not admit new residents requiring palliative care due to lack of resources and trained staff…. Approximately one third of the DONs indicated they had transferred or discharged a resident requiring palliative care to another institution or the community, 76% to an acute hospital, 14% to hospice and 8% to the community (i.e., home, homeland or a different city)			2. Recognition of gaps and needs
	The majority of DONs (69%) had attended an educational course on palliative care, however, only 16% had formal palliative care qualifications.			3. Competence in fulfilling the complex responsibilities
	Only a small number of DONs (18%) indicated there were no barriers to providing palliative care in their nursing home. It was believed by the majority of DONs that present educational resources pn palliative care were inadequate			2. Recognition of gaps and needs
	The majority expressed various barriers:	Lack of resources 71% DONs (lack of education and training for all nursing home staff, general practitioners, and volunteers)		2. Recognition of gaps and needs
		Inadequate eduation 47% (a lack of education and training for all nursing home staff, general practitioners, and volunteers)		2. Recognition of gaps and needs
		Environment factors 28% (such as unsuitable buildings, structural issues, lack of single rooms/space, and no privacy for residents and families and wandering residents causing problems)		2. Recognition of gaps and needs
		Professional conflicts 18%: The majority of DONs (63%) reported having experienced differences of opinion between professionals in the appropriate management of palliative care residents and most gave example.		3. Competence in fulfilling the complex responsibilities
	The palliative care support utlised by DONs (81%) centred on some form of palliative care expertise, consultancy, or support			2. Recognition of gaps and needs
	19% had not utilized any form of palliative care support. Reasons for not utilizing palliative care support included: the nursing home having specialists who had experience and/or qualifications in palliative care; Palliative care beds being officially designated and recognized; the need not arising; and no access to palliative care support.			2. Recognition of gaps and needs
	The majority of DONs (78%) felt that they did not have access to hospice beds if required, and of the 22% who stated they had access, only 15% had actually utilized this option.			2. Recognition of gaps and needs
	The majority of DONs (60%) indicated the nursing home had a formal palliative care policy, but bereavement work with staff and relatives was on an informal and on an as‐needed basis. This informal grief work included: Counseling of residents, families, and staff Pastoral care and memorial services Funeral attendance by staff Staff involvement with family, reminiscence, debriefing and discussion Books/booklets on grief			2. Recognition of gaps and needs
Eriksson et al. (2015)	Of the MAs, 50% (*n* = 22) attended, and 33% (*n* = 14) of them lacked education		50% managers had attended palliative care workshop and 33% managers of them lacked palliative care education (around 64.4%)	3. Competence in fulfilling the complex responsibilities
	A total of 50% of MAs (*n* = 17) needed further education.			3. Competence in fulfilling the complex responsibilities
	14% of the MAs were in need of support of counseling specialists in palliative care outside the care setting (*n* = 6).			3. Competence in fulfilling the complex responsibilities
Froggatt and Payne (2006)	Sixty‐one managers (75%) provided a definition for end‐of‐life care and the responses encompassed both living in the care home and the final acts of care provided for residents once they had died.			1. Conceptual grasp of palliative care
	For five managers end‐of‐life care had a broad remit		For 6.2% of managers end‐of‐life care had a broad remit	1. Conceptual grasp of palliative care
	Living and life was also seen by nine managers as being a part of this domain		Living and life was also seen by 11.1% managers as being a part of this domain	1. Conceptual grasp of palliative care
	Managers of 73 homes (90%) describe the provision of end‐of‐life care at different time periods, the focal point of end‐of‐life care is the death event.			1. Conceptual grasp of palliative care
	Internal resources: When asked if their staff had received any training about the care of dying residents, 63% (*n* = 51) of managers indicated that this had occurred			2. Recognition of gaps and needs
	Internal resources: A great proportion of nursing homes (82%) provided staff training in provision of end of life care			2. Recognition of gaps and needs
	Internal resources: Nearly all managers (98%) indicated that their home had a fan and nearly all managers (91%) indicated that their home had equipment for pressure relief; in 80% homes there were refreshment facilities for family and a quite room; the equipment least available was specific to palliative care practice such as a syringe driver (16%) or a TENS (15%) machine for pain relief			2. Recognition of gaps and needs
	Relationships with external providers of care: 65% managers (*n* = 44) indicated that the home had access to 24‐h palliative care support.			2. Recognition of gaps and needs
Mitchell et al. (2016)	‐ Successfully answered items: items 1, 4, 9, 11, 15 which were all correctly answered by more than 85% of participants. There are several key themes within these items; the appropriateness of palliative care, pain, and grief. The high number of correct responses suggest that care home managers are generally aware of the broader uses of palliative care prior to the end‐of‐life stages, there is a strong knowledge base regarding the use of therapeutic pain relieving strategies, and the emotional and psychological aspects of care including the roles of emotion and grief.		More than 85% correction: Items 1, 4, 9, 11, 15 covered palliative care appropriateness, pain management strategies, and grief/emotional aspects of care	1. Conceptual grasp of palliative care 3. Competence in fulfilling the complex responsibilities
	‐ Poorly answered items: questions 2, 6, 10, 12, and 20 were answered correctly by less than 50% of participants. While the items correctly answered more general topics such as pain and social aspects of palliative care, the poorly answered items are focused on the biological and physical components of care, for example electrolyte imbalances and respiratory depression. Additionally, question 12 may be controversial as the focus of palliative care is that of caring over curing, however, the underlying philosophy of palliative care includes the best possible quality of life, alongside the management of all physical, psychological, social, and spiritual symptoms, making it comparable to an aggressive treatment. This confusion may explain why 75% of participants answered this question incorrectly		Less than 50% correct: items 2, 6, 10, 12, 20 focused on biological and physiological aspects of palliative care, such as electrolyte imbalances and respiratory depression	1. Conceptual grasp of palliative care
	Additionally, question 12 may be controversial as the focus of palliative care is that of caring over curing, however, the underlying philosophy of palliative care includes the best possible quality of life, alongside the management of all physical, psychological, social, and spiritual symptoms, making it comparable to an aggressive treatment. This confusion may explain why 75% of participants answered this question incorrectly		A confusion of Item 12 (the underlying philosophy of palliative care) with 75% answered incorrectly	1. Conceptual grasp of palliative care
	‐ Items eliciting a ‘don't know’ response: For items 2, 10, and 16, over 10% of participants responded with ‘don't know’, suggesting confusion around the area of pain. The topic of pain is central to many of the questions in the survey, and these questions have a wide range in response rates from almost everyone answering correctly to the majority answering incorrectly.		Over 10% don't know: Items 2, 10, and 16 in relation to pain management	1. Conceptual grasp of palliative care
Tunnard et al. (2024)	Over 76% of survey respondents sought palliative and end‐of‐life care advice from GPs (79.2% of nursing, 79.4% of dual)			2. Recognition of gaps and needs
	Most care homes with on‐site registered nurses sought palliative and end‐of‐life care support from specialist palliative care services (92% nursing and 88% dual)			2. Recognition of gaps and needs
	All nursing home, 88% of dual and 63% of residential home respondents reported using guidance to deliver palliative and end‐of‐life care (additional file 3). Guidance varied from large, well‐embedded initiatives, for example the Gold Standards Framework, to in‐house guidance. Developed by experienced staff.			2. Recognition of gaps and needs

#### 2.5.2 Exploring Relationships in the Data

The qualitative themes and quantitative descriptive results were compared by contextualizing the quantitative findings within qualitative narratives and explanations (Popay et al. 2006). This process involved examining similarities and discrepancies to integrate both data types into a coherent interpretation. When the data from multiple studies showed similarities, reciprocal translation was conducted to complement and synthesize the findings. When the data showed contradictions, refutational translation was conducted to compare and contrast the findings (Popay et al. 2006).

#### 2.5.3 Assessing the Robustness of the Synthesis

The synthesis process was collaboratively conducted by the first and last authors and was continuously reviewed and validated by the other authors. The iterative nature of the synthesis allowed for critical evaluation, refinement of themes, and resolution of discrepancies through ongoing dialogue and consensus among all authors (Popay et al. 2006). The quality appraisal of all included studies was conducted and considered throughout the synthesis and interpretation of findings.

## 3 Results

In total, eight articles presenting findings from eight studies were included in the analysis (Bükki et al. 2016; De Bellis and Parker 1998; Eriksson et al. 2015; Froggatt and Payne 2006; Katz et al. 2000; Mitchell et al. 2016; Tunnard et al. 2024; Arjama et al. 2025). Among these, three studies were conducted in the United Kingdom (Froggatt and Payne 2006; Katz et al. 2000; Tunnard et al. 2024), while the remaining studies were conducted in Germany (Bükki et al. 2016), Australia (De Bellis and Parker 1998), Sweden (Eriksson et al. 2015), Ireland (Mitchell et al. 2016), and Finland (Arjama et al. 2025). These studies utilized different designs, including qualitative (Bükki et al. 2016; Katz et al. 2000; Arjama et al. 2025), quantitative (De Bellis and Parker 1998; Eriksson et al. 2015; Mitchell et al. 2016), and mixed‐methods designs (Froggatt and Payne 2006; Tunnard et al. 2024). Qualitative studies collected data using focus group interviews, semi‐structured individual interviews, and observations (Bükki et al. 2016; Tunnard et al. 2024; Arjama et al. 2025). Quantitative studies collected data using questionnaires containing closed‐ and open‐ended questions (De Bellis and Parker 1998; Froggatt and Payne 2006; Katz et al. 2000). Mixed‐methods studies used questionnaires, interviews, and participant observations to collect data (Bükki et al. 2016; Froggatt and Payne 2006; Mitchell et al. 2016; Tunnard et al. 2024). The number of NMs explicitly reported in the included studies was 436, whereas some studies did not specify the exact number of NMs among their participants (Bükki et al. 2016; Tunnard et al. 2024). The settings that the studies focused on included nursing homes, residential care homes, home care services, and group residential settings. The quality of the studies ranged from low to high, with three studies classified as low quality (De Bellis and Parker 1998; Froggatt and Payne 2006; Katz et al. 2000), three studies as moderate quality (Bükki et al. 2016; Mitchell et al. 2016; Tunnard et al. 2024), and two studies as high quality (Eriksson et al. 2015; Arjama et al. 2025). According to the JBI Critical Appraisal Checklist for Qualitative Research, the limitations of the included qualitative studies were mainly that they had implicit philosophical perspectives (Bükki et al. 2016; Katz et al. 2000; Arjama et al. 2025), an inconsistent aim (Bükki et al. 2016), insufficient or unclear descriptions of methodology (Katz et al. 2000), incongruity between the analysis and results presentation (Bükki et al. 2016; Katz et al. 2000), unstated researchers' influence (Bükki et al. 2016; Katz et al. 2000), and unstated ethical considerations (Katz et al. 2000). The study lacking ethical statements was not excluded, because the research focus and population were not considered sensitive. According to the AXIS, the limitations of the included quantitative studies were mainly that the studies did not demonstrate a clear definition of the target population (De Bellis and Parker 1998), an appropriate representative population (Mitchell et al. 2016), descriptions of questionnaire development or modifications (De Bellis and Parker 1998; Mitchell et al. 2016), statistical significance due to descriptive statistics (De Bellis and Parker 1998; Mitchell et al. 2016), or demographic data on population (De Bellis and Parker 1998; Mitchell et al. 2016), and that they had a low response rate (De Bellis and Parker 1998). Additionally, concerns were raised regarding the internal consistency of the results (De Bellis and Parker 1998; Mitchell et al. 2016). According to the MMAT, the limitations of included mixed‐methods studies resided primarily in their quantitative components and the integration of mixed methods. These limitations included sampling strategies that were not considered relevant to address the research question (Tunnard et al. 2024), inadequate descriptions of the development or modification of the measurement tool (Froggatt and Payne 2006), unaddressed response rates (Froggatt and Payne 2006; Tunnard et al. 2024), a lack of adequate rationale for using a mixed‐methods design (Froggatt and Payne 2006; Tunnard et al. 2024), and an inadequate integration of qualitative and quantitative components (Froggatt and Payne 2006).

### 3.1 Narrative Synthesis

The narrative synthesis resulted in three main themes: (1) Conceptual grasp of palliative care, (2) Recognition of gaps and needs, and (3) Competence in fulfilling complex responsibilities.

#### 3.1.1 Conceptual Grasp of Palliative Care

Various insights into NMs' perceptions of the palliative approach as integrated into nursing homes in municipal care indicated their understanding of the underlying philosophy and principles of PC (Froggatt and Payne 2006; Tunnard et al. 2024). Nurse managers recognized several core principles of a palliative approach, including the importance of relief from suffering, of person‐centredness, and of a holistic care perspective that addressed physical, psychological, emotional, cultural, and spiritual needs (De Bellis and Parker 1998; Mitchell et al. 2016; Arjama et al. 2025). Overall, NMs generally acknowledged the value of PC and the importance of integrating it into municipal care (Bükki et al. 2016; De Bellis and Parker 1998; Katz et al. 2000; Mitchell et al. 2016; Tunnard et al. 2024). However, there were variations in NMs' understanding of the PC concept and principles, in terms of both theoretical comprehension and practical application of the palliative approach to care (Bükki et al. 2016; De Bellis and Parker 1998; Froggatt and Payne 2006; Katz et al. 2000; Mitchell et al. 2016; Tunnard et al. 2024). For example, many NMs associated PC only with end‐of‐life care, demonstrating a lack of awareness of its broader application across the illness trajectory (Froggatt and Payne 2006), although some did recognize the broader application of PC beyond the end‐of‐life stage (Mitchell et al. 2016). It was also noted by NMs that the term “palliative” was often oversimplified in practice, sometimes applied based merely on patients' refusal to transfer to hospital rather than a holistic assessment aligned with the principles of a palliative approach (Bükki et al. 2016). When NMs' knowledge was evaluated through a PC quiz, it was found that while NMs had a good knowledge base with regard to the psychosocial components of PC, their knowledge and understanding of the physical aspect, especially symptom management of, for example, dyspnoea and pain, were limited (Mitchell et al. 2016). On the other hand, some NMs were able to provide a definition of PC that was grounded in its philosophy and aligned with its core principles, showing that they had a solid understanding of the PC concept (De Bellis and Parker 1998). Nurse managers emphasized the importance of person‐centredness in care in terms of understanding and respecting patients' and relatives' needs and preferences in the final stage of life (Bükki et al. 2016; Froggatt and Payne 2006; Arjama et al. 2025). Consistent and detailed communication with relatives about care decisions and information was perceived by NMs as a key element in ensuring person‐centredness in PC (Bükki et al. 2016; Froggatt and Payne 2006; Arjama et al. 2025).

#### 3.1.2 Recognition of Gaps and Needs

Nurse managers were able to identify gaps in their knowledge and practice that affected their confidence in PC and to recognize the conditions required for developing their competence in this area (Bükki et al. 2016; De Bellis and Parker 1998; Froggatt and Payne 2006; Katz et al. 2000; Tunnard et al. 2024). This was a process driven by their conceptual understanding of PC and their experiences of how such awareness revealed existing gaps between their expectations and the realities of practices, thus highlighting the need to bridge the gaps (Bükki et al. 2016; De Bellis and Parker 1998; Froggatt and Payne 2006; Katz et al. 2000; Tunnard et al. 2024; Arjama et al. 2025).

Nurse managers viewed having guidelines and policies for standardizing the care delivery and ensuring adequate resources to support their teams as essential for both increasing their confidence in PC and supporting the quality of PC practice (Bükki et al. 2016; De Bellis and Parker 1998; Froggatt and Payne 2006; Katz et al. 2000; Tunnard et al. 2024; Arjama et al. 2025). The guidelines and policies highlighted included those related to areas where they identified gaps and barriers that affected the overall quality of care, such as care planning and documentation (Eriksson et al. 2015; Katz et al. 2000; Tunnard et al. 2024), identification of the course of illness (Tunnard et al. 2024), and involvement and support of relatives in care (De Bellis and Parker 1998; Katz et al. 2000). Moreover, NMs recognized the importance of education and training, resources in terms of sufficient and appropriate staffing, and infrastructure and equipment within the care settings (Bükki et al. 2016; Froggatt and Payne 2006; Katz et al. 2000). Lack of resources exacerbated the gaps in PC practices in municipal care, limiting not only the provision of care but also NMs' confidence and readiness for PC as well as the conditions for developing PC competence (Bükki et al. 2016; De Bellis and Parker 1998; Froggatt and Payne 2006; Katz et al. 2000; Tunnard et al. 2024). Some NMs identified that public discourse about PC in society was insufficient, which they perceived as an influential factor affecting resource priorities and allocation within the healthcare system (Arjama et al. 2025).

Some NMs in nursing homes found that resource allocation varied across different care settings (Bükki et al. 2016; De Bellis and Parker 1998; Froggatt and Payne 2006; Katz et al. 2000; Tunnard et al. 2024). Their experience was that nursing homes often had fewer resources than hospitals, particularly in terms of personnel and knowledge resources, including guidelines, facilities, and equipment (Bükki et al. 2016; De Bellis and Parker 1998; Froggatt and Payne 2006; Katz et al. 2000; Tunnard et al. 2024; Arjama et al. 2025). The lack of resources can give rise to ethical challenges (Arjama et al. 2025), resulting in increased dependence on external support, such as a specialist PC team, for knowledge, equipment, and assistance in meeting patients' needs and ensuring appropriate care (Bükki et al. 2016; De Bellis and Parker 1998; Froggatt and Payne 2006; Katz et al. 2000; Tunnard et al. 2024). Some NMs considered the access to a specialist PC team as a means to bridge the gap in care quality between municipal care and hospital care (Tunnard et al. 2024). Others, recognizing the lack of PC competence and the gaps in PC practices, expressed a strong need for specialist PC teams' support in order to build confidence in providing PC and meeting current PC needs (Eriksson et al. 2015).

#### 3.1.3 Competence in Fulfilling the Complex Responsibilities

There was a close link between NMs' competence in PC and their ability to perform the complex responsibilities that their roles entailed, confidence acting as both a reflection and a reinforcement of their competence (De Bellis and Parker 1998; Katz et al. 2000; Arjama et al. 2025). Nurse managers held complex and multifaceted roles in PC provision, including interacting with patients and relatives, prioritizing multiple tasks and responsibilities, providing emotional and knowledge support for RNs, collaborating with other professionals, such as doctors and specialist PC teams, and identifying quality deficiencies in care (Bükki et al. 2016; Eriksson et al. 2015; Froggatt and Payne 2006; Tunnard et al. 2024; Arjama et al. 2025).

Nurse managers stressed the importance of PC education, knowledge, and work experience in building their confidence and ability in delivering, assessing, prioritizing care, as well as in making ethically appropriate decisions related to PC (Eriksson et al. 2015; Arjama et al. 2025). Lack of education in PC made it difficult for NMs to engage in care‐related discussions, resolve care‐related disagreements among RNs, and provide support to RNs (Eriksson et al. 2015). Although many NMs had attended some form of PC education, they perceived that their knowledge of PC was still insufficient for them to confidently fulfill their roles, and they thus expressed a strong need for further formal PC training (Froggatt and Payne 2006; Katz et al. 2000). Additionally, NMs' confidence and sense of professional resilience were closely associated with RNs' competence in providing PC (De Bellis and Parker 1998; Froggatt and Payne 2006; Katz et al. 2000).

Furthermore, the involvement of patients and their relatives in care planning and decision‐making was emphasized by many NMs, who recognized it as significant for person‐centred care and as a key aspect of their leadership responsibility (Froggatt and Payne 2006; Tunnard et al. 2024; Arjama et al. 2025). Many NMs actively engaged in assessing the quality of care and acknowledged their responsibility to understand the needs of patients and their relatives in order to fulfill this role (Bükki et al. 2016; Katz et al. 2000; Arjama et al. 2025). They reminded RNs to remain attentive to the needs of patients and their relatives in care and, at times, identified the need to provide practical and emotional support to patients and their relatives themselves (Bükki et al. 2016; Froggatt and Payne 2006; Katz et al. 2000; Arjama et al. 2025). However, NMs expressed concerns about the ethical challenges that arose in PC, particularly at the end‐of‐life stage (Bükki et al. 2016; Arjama et al. 2025). They reported conflicts between patients' and relatives' preferences in care decisions, where some NMs experienced difficulties in communicating and navigating these situations (Bükki et al. 2016; Arjama et al. 2025).

Nurse managers had gained knowledge of and skills in PC with the support of specialist PC teams, through their regular visits, guidance on complex cases, and professional training, which strengthened NMs' competence and enhanced their confidence in handling complex situations (Bükki et al. 2016; Eriksson et al. 2015; Froggatt and Payne 2006; Tunnard et al. 2024). Some NMs were also able to provide emotional support to RNs through facilitating open discussions about death and dying within the team, which created a supportive environment in the care settings and, in turn, strengthened NMs' own sense of readiness and confidence in providing care (Bükki et al. 2016; Froggatt and Payne 2006; Katz et al. 2000). Notably, NMs perceived the support, such as consultation, resources, and availability, from the senior management as crucial for performing their leadership responsibilities ethically (Arjama et al. 2025).

## 4 Discussion

This systematic review synthesized the current evidence on NMs' awareness of and competence in PC in municipal care settings. The results showed a lack of sufficient evidence in the current area of study, highlighting the need for further research. Based on the limited data, the results suggested that NMs' conceptual understanding and their interpretation of PC shaped their practical focus and priorities as well as their recognition of areas for improvement in relation to PC. This awareness played a key role in shaping their perceived competence, partially through its influence on their confidence. The results further indicated that NMs' competence in PC was not only diverse but also closely tied to their confidence in leading complex care processes, which in turn affected their ability to support RNs and ensure high‐quality PC.

The results of the current review suggested that NMs' awareness of PC often shaped their recognition of the PC approach and their ability to identify gaps in, and set priorities for, competence development in their everyday practices. This aligns with the theory of consciousness, in that awareness involves a conscious perception of, and the capacity to reliably articulate and reflect on one's own experiences (Rosenthal 2019; Chun and Johnson 2011). The findings that NMs often lacked a clear understanding of the PC concept and principles are consistent with previous research (Pereira et al. 2017; Wallerstedt et al. 2019), which shows that other municipal care managers similarly recognized the value of PC and its role in promoting quality of life, yet frequently demonstrated limited comprehension of its core principles and the full scope of care it entails. Such conceptual uncertainty and inadequacy may lead to barriers to integrating a PC approach into routine practices, often in ways that NMs may not fully recognize or be aware of (Wallerstedt et al. 2019; Mahon and McAuley 2010). Although NMs were able to recognize the areas of improvement in care practices based on the current results, their ability to support RNs and cooperate with the municipality responsible for resource allocation was also important in addressing the gaps and challenges (Sawatzky et al. 2016; Miltner et al. 2015). Previous studies have reported considerable variation in the implementation of PC guidelines and their outcomes (Robinson et al. 2023; Sawatzky et al. 2017). A contributing factor may relate to the limited evidence base on how PC should be integrated within municipal care settings, combined with NMs' varying levels of PC knowledge and organizational support (Wilkinson et al. 2011). Given NMs' leadership role in shaping the caring culture in municipal care settings (World Health Organisation 2018), their awareness of PC is reflected across all aspects of their work, particularly in how and where they direct their attention to cultivating a palliative approach within their care setting (Webb et al. 2016). Nurse managers who set a shared vision of care within their teams, align care with the best available evidence, and take risks while seizing opportunities to create accessible, equitable, and sustainable PC often strategically and systematically support and facilitate RNs to strengthen their capacity and enhance collaboration in integrating a palliative approach into everyday practices (Phillips and Agar 2016). Nurse managers' leadership therefore plays a key role in not only supporting competence development but also shaping a care culture where palliative principles are embedded and consistently practiced in decision‐making. Often, this care culture is also characterized by openness to change and innovation informed by the best available evidence (Phillips and Agar 2016; Nilsen et al. 2018). Accordingly, NMs' competence in PC is built on their appropriate awareness, which shapes their attention and contributes to effective leadership in delivering high‐quality care and identifying opportunities for competence development.

The current results further indicated that NMs' varied levels of competence in PC affected their engagement in care delivery and their performances of complex responsibilities, often reflected in their self‐perceived confidence when facing specific tasks in relation to PC. In line with previous research (González‐García et al. 2021; Silva et al. 2022), healthcare knowledge and competence in general are fundamental for NMs to effectively perform their role. As awareness shapes one's confidence in understanding and responding to a given task or situation, NMs' confidence regarding PC, grounded in such awareness, may indicate how well awareness and competence are aligned with respect to specific tasks related to PC (Rosenthal 2019; Gottlieb et al. 2022). Their awareness also reflects NMs' readiness to engage in PC as well as functioning as a determinant of performance in leadership roles (Gottlieb et al. 2022; Owens and Keller 2018; Bandura 1977). Studies have shown that NMs who demonstrate confidence in PC can foster a supportive environment where RNs feel more empowered and competent to provide holistic, person‐centred PC (Bandura 1982; Van Dyk et al. 2016). According to social cognitive theory (Bandura 1982, 1986), individuals' cognition and other personal factors, individuals' behavior and the performance environment are dynamically interrelated and mutually influence one another. Within this framework, a leader's cognition, or self‐efficacy, can be understood as their belief in their ability to effectively perform leadership tasks (McCormick 2001). Chemers et al. (2000) argued that self‐confidence influences leadership performance through the mediating role of leadership self‐efficacy. In other words, NMs' confidence in their capabilities shapes their self‐efficacy beliefs, which in turn guide their behavioral intentions and performances in leadership practice related to PC. Thus, NMs' awareness serves as a foundational element, whereas their confidence, as mentioned above, acts as a mediator in how awareness and competence are aligned and function in practice.

Furthermore, the complex responsibilities in NMs' role have been confirmed by other studies (Van Dyk et al. 2016; Miltner et al. 2015). Their role entails not only care delivery and coordination but also a wide range of managerial tasks that often dominate their daily work, in which their competence in PC is also embedded (Van Dyk et al. 2016; Miltner et al. 2015; Wilkinson et al. 2011). Nurse managers in municipal care settings are often expected to balance the ideal standards of person‐centred, high‐quality care with the realities of limited resources and support, while remaining accountable for care outcomes (McGregor 2008; Lugtenberg et al. 2009; Andrews and Dollery 2021). Challenges may arise from the gap between the expectations and the realities in practice, which may not only limit NMs' opportunities to develop PC competence but affect their confidence in terms of their self‐perceived ability to effectively influence care practice. Accordingly, NMs' PC competence is suggested to emerge through a dynamic interplay of individual attributes (e.g., knowledge, confidence) and organizational conditions (e.g., resources, support structures). Given that municipal care is a central setting for general PC, the gap in the current literature, where limited attention has been given to NMs in relation to PC in municipal care, may contribute to a lack of understanding and support for developing the PC competence required for NMs in municipal care (Van Dyk et al. 2016; Perez‐Gonzalez et al. 2024). Future research is thus needed to deepen the understanding of NMs' competence in PC and to explore the PC competence they need in their leadership role in municipal care.

## 5 Limitations

This review had some limitations. Firstly, the review included only studies published in English. Potentially relevant data in literature published in other languages may be overlooked. Secondly, NMs in different countries have varying qualifications and roles, which may lead to variations in the implementation and understanding of PC and in NMs' support of RNs. These variations may make it more difficult to compare results across countries and interpret how NMs influence PC practices. Thirdly, only eight studies were assessed as relevant for inclusion in this review, with only one study including NMs in home care settings (Eriksson et al. 2015). The database search retrieved a large number of records due to the exhaustive and sensitive search strategy. The inclusion criteria did not restrict search based on publication date. However, most records were excluded because they did not meet the context, population, or outcome criteria, leading to only a small number of included articles. This can be explained by the lack of available knowledge and evidence within the review scope. Additionally, intervention studies were excluded from the analysis because the current review did not intend to evaluate specific interventions. Gray literature was not searched, which may have led to the risk of publication bias. However, gray literature is often excluded from systematic reviews due to variability in quality and limited peer review. Consequently, relevant data from these sources may not have been captured in this review. The limited data may affect the generalisability of the results of the review. Fourthly, the systematic steps of the narrative synthesis ensure the transparency and rigor of the synthesis (Morrison et al. 2022). However, the absence of the theory development step may influence the depth of interpretation and potentially limit the synthesis from moving beyond the descriptive level. Lastly, the inclusion of low‐quality studies may have influenced the overall confidence of the results. Nevertheless, data from low‐quality studies, as confirmed by previous research (Lund et al. 2021), were still considered to contribute relevant knowledge and insights into this under‐researched area and may, more importantly, inform the direction of future research.

## 6 Conclusion

This systematic review compiled existing evidence on awareness and competence in PC among NMs in municipal care settings. The results pointed to a significant knowledge gap in this area. However, the current evidence base was limited, comprising only a small number of studies of varying quality. The results highlighted a call for future research using diverse methods and perspectives to provide high‐quality, contextually relevant knowledge about NMs' awareness of and competence in PC and about how these aspects are integrated into NMs' everyday practices in municipal care. Future research needs to explore NMs' perspectives on how they understand and perform their leadership role within the interactions, collaboration and relationships in relation to PC, in order to understand the complexity of leadership in PC in municipal care settings. Additionally, NMs' confidence or self‐efficacy, as an important mediator in understanding their leadership behaviors and practices in PC, should be taken into account in future research.

## Author Contributions

**Hongxuan Xu:** conceptualization, methodology, investigation, validation, formal analysis, writing – original draft, writing – review and editing, data curation, software, funding acquisition, visualization, resources. **Marie‐Louise Möllerberg:** conceptualization, methodology, data curation, supervision, formal analysis, validation, investigation, writing – original draft, writing – review and editing, software, funding acquisition, visualization, resources. **Karin Dalhammar:** conceptualization, methodology, data curation, supervision, formal analysis, validation, investigation, writing – original draft, writing – review and editing, software, funding acquisition, visualization, resources. **Mariette Bengtsson:** conceptualization, methodology, data curation, supervision, formal analysis, validation, investigation, writing – original draft, writing – review and editing, software, funding acquisition, visualization, resources. **Katarina Sjögren Forss:** conceptualization, methodology, data curation, supervision, formal analysis, validation, investigation, writing – original draft, writing – review and editing, software, funding acquisition, visualization, resources, project administration.

## Funding

This work was supported by Foundation for the Promotion of Malmö University's Development.

## Disclosure

Understanding nurse managers' awareness and competence in palliative care can provide insight into how palliative care leadership influences clinical practice, particularly by mean of ensuring care quality, supporting registered nurses, and facilitating collaboration in relation to palliative care. Furthermore, it highlights the importance of palliative care knowledge among nurse managers in effectively fulfilling their leadership roles and contributing to high‐quality care. Overall, the findings highlight the need for a deeper understanding of nurse managers' leadership role in palliative care, as well as continued development of leadership competence in palliative care.

## Ethics Statement

The authors have nothing to report.

## Consent

The authors have nothing to report.

## Conflicts of Interest

The authors declare no conflicts of interest.

## Supporting information


**File S1:** PRISMA checklist.

**File S2:** Search strategy.

## Data Availability

The data that support the findings of this study are available from the corresponding author upon reasonable request.

## References

[nhs70352-bib-0001] Adams, L. 2011. “Learning a New Skill Is Easier Said Than Done.” Gordon Training International.

[nhs70352-bib-0002] Albers, G. , S. Pereira , B. Onwuteaka‐Philipspen , L. Deliens , R. Pasman , and L. Van den Block . 2015. “A Public Health Perspective on Palliative Care for Older People: An Introduction.” In Palliative Care for Older People: A Public Health Perspective, edited by L. A. G. Van den Block , S. Pereira , S. M. Pereira , et al., 3–15. Oxford University Press.

[nhs70352-bib-0003] Amankwah, O. , W. W. Choong , and N. A. Boakye‐Agyeman . 2022. “The Relationship Between Facilities Management Service Quality and Patients' Health‐Care Experience: The Mediating Effect of Adequacy of Health‐Care Resource.” Facilities 41, no. 1/2: 108–125.

[nhs70352-bib-0004] Andersson, H. , M. Lindholm , M. Pettersson , and L. L. Jonasson . 2017. “Nurses' Competencies in Home Healthcare: An Interview Study.” BMC Nursing 16: 65. 29176934 10.1186/s12912-017-0264-9 PMC5693583

[nhs70352-bib-0005] Andrews, R. , and B. Dollery . 2021. “Guest Editors' Introduction: The Impact of Ageing and Demographic Change on Local Government.” Local Government Studies 47, no. 3: 355–363.

[nhs70352-bib-0006] Arjama, A. L. , R. Suhonen , and M. Kangasniemi . 2025. “Ethical Issues Encountered by Nurse Managers Working With Older Adults in Long‐Term Care Settings: A Qualitative Interview Study.” Journal of Nursing Management 2025: 3978256. 40401040 10.1155/jonm/3978256 PMC12094859

[nhs70352-bib-0007] Aromataris, E. , K. Porritt , and B. Pilla , eds. 2024. JBI Manual for Evidence Synthesis. JBI. 10.46658/JBIMES-24-01.

[nhs70352-bib-0008] Arvidsson, B. , and B. Fridlund . 2005. “Factors Influencing Nurse Supervisor Competence: A Critical Incident Analysis Study.” Journal of Nursing Management 13, no. 3: 231–237. 15819835 10.1111/j.1365-2834.2004.00532.x

[nhs70352-bib-0009] Asante, B. L. , F. Zúñiga , and L. Favez . 2021. “Quality of Care Is What We Make of It: A Qualitative Study of Managers' Perspectives on Quality of Care in High‐Performing Nursing Homes.” BMC Health Services Research 21, no. 1: 1090. 34645441 10.1186/s12913-021-07113-9 PMC8515763

[nhs70352-bib-0010] Bandura, A. 1977. “Self‐Efficacy: Toward a Unifying Theory of Behavioral Change.” Psychological Review 84, no. 2: 191–215. 847061 10.1037//0033-295x.84.2.191

[nhs70352-bib-0011] Bandura, A. 1982. “Self‐Efficacy Mechanism in Human Agency.” American Psychologist 37, no. 2: 122–147.

[nhs70352-bib-0012] Bandura, A. 1986. Social Foundations of Thought and Action: A Social Cognitive Theory. Prentice‐Hall, Inc.

[nhs70352-bib-0013] Batt, A. M. , W. Tavares , and B. Williams . 2020. “The Development of Competency Frameworks in Healthcare Professions: A Scoping Review.” Advances in Health Sciences Education: Theory and Practice 25, no. 4: 913–987. 31797195 10.1007/s10459-019-09946-w

[nhs70352-bib-0014] Becker, R. 2007. “The Development of Core Competencies for Palliative Care Educators.” International Journal of Palliative Nursing 13, no. 8: 377–383. 18018817 10.12968/ijpn.2007.13.8.24536

[nhs70352-bib-0015] Becker, R. 2010. Fundamental Aspects of Palliative Care Nursing. An Evidence‐Based Handbook for Student Nurses. Quay Books Division, MA Healthcare Ltd.

[nhs70352-bib-0016] Bükki, J. , P. M. Neuhaus , and P. Paal . 2016. “End of Life Care in Nursing Homes: Translating Focus Group Findings Into Action.” Geriatric Nursing 37, no. 6: 440–445. 27406626 10.1016/j.gerinurse.2016.06.011

[nhs70352-bib-0017] Burrell, L. V. , H. M. Rostad , T. Wentzel‐Larsen , M. S. Skinner , and M. K. R. Sogstad . 2023. “The Influence of Individual and Municipality Characteristics on Allocation of Long‐Term Care Services: A Register‐Based Cross‐Sectional Study.” BMC Health Services Research 23, no. 1: 801. 37501173 10.1186/s12913-023-09641-y PMC10373409

[nhs70352-bib-0018] Chandler, J. , M. Cumpston , T. Li , M. J. Page , and V. J. Welch , eds. 2019. Cochrane Handbook for Systematic Reviews of Interventions. 2nd ed. John Wiley & Sons.

[nhs70352-bib-0019] Chemers, M. M. , C. B. Watson , and S. T. May . 2000. “Dispositional Affect and Leadership Effectiveness: A Comparison of Self‐Esteem, Optimism, and Efficacy.” Personality and Social Psychology Bulletin 26, no. 3: 267–277.

[nhs70352-bib-0020] Chun, M. M. , and M. K. Johnson . 2011. “Memory: Enduring Traces of Perceptual and Reflective Attention.” Neuron 72, no. 4: 520–535. 22099456 10.1016/j.neuron.2011.10.026 PMC3248396

[nhs70352-bib-0021] Cohen, E. R. , J. H. Barsuk , W. C. McGaghie , and D. B. Wayne . 2013. “Raising the Bar: Reassessing Standards for Procedural Competence.” Teaching and Learning in Medicine 25, no. 1: 6–9. 23330888 10.1080/10401334.2012.741540

[nhs70352-bib-0022] Cooke, A. , D. Smith , and A. Booth . 2012. “Beyond PICO: The SPIDER Tool for Qualitative Evidence Synthesis.” Qualitative Health Research 22, no. 10: 1435–1443. 22829486 10.1177/1049732312452938

[nhs70352-bib-0089] Covidence Systematic Review Software . 2025. “Veritas Health Innovation.” Melbourne, Australia. Available at www.covidence.org.

[nhs70352-bib-0023] Cowan, D. T. , I. Norman , and V. P. Coopamah . 2007. “Competence in Nursing Practice: A Controversial Concept—A Focused Review of Literature.” Accident and Emergency Nursing 15, no. 1: 20–26. 17276294 10.1016/j.aaen.2006.11.002

[nhs70352-bib-0024] De Bellis, A. , and D. Parker . 1998. “Providing Palliative Care in Australian Nursing Homes: Issues and Challenges.” Geriaction 16, no. 3: 17–23.

[nhs70352-bib-0025] Downes, M. J. , M. L. Brennan , H. C. Williams , and R. S. Dean . 2016. “Development of a Critical Appraisal Tool to Assess the Quality of Cross‐Sectional Studies (AXIS).” BMJ Open 6, no. 12: e011458. 10.1136/bmjopen-2016-011458 PMC5168618 27932337

[nhs70352-bib-0026] Dwyer, D. 2011. “Experiences of Registered Nurses as Managers and Leaders in Residential Aged Care Facilities: A Systematic Review.” International Journal of Evidence‐Based Healthcare 9, no. 4: 388–402. 22093388 10.1111/j.1744-1609.2011.00239.x

[nhs70352-bib-0027] Eriksson, G. , T. W. Bergstedt , and C. Melin‐Johansson . 2015. “The Need for Palliative Care Education, Support, and Reflection Among Rural Nurses and Other Staff: A Quantitative Study.” Palliative & Supportive Care 13, no. 2: 265–274. 24576441 10.1017/S1478951513001272

[nhs70352-bib-0028] Franco, A. , M. T. C. Vidigal , M. N. Oliveira , C. T. J. S. Nascimento , R. F. Silva , and L. R. Paranhos . 2020. “Evidence‐Based Mapping of Third Molar Techniques for Age Estimation Applied to Brazilian Adolescents—A Systematic Review.” Research, Society and Development 9, no. 10: e9339109395.

[nhs70352-bib-0029] Froggatt, K. , and S. Payne . 2006. “A Survey of End‐Of‐Life Care in Care Homes: Issues of Definition and Practice.” Health & Social Care in the Community 14, no. 4: 341–348. 16787485 10.1111/j.1365-2524.2006.00628.x

[nhs70352-bib-0030] González‐García, A. , A. Pinto‐Carral , S. Pérez‐González , and P. Marqués‐Sánchez . 2021. “Nurse Managers' Competencies: A Scoping Review.” Journal of Nursing Management 29, no. 6: 1410–1419. 34018273 10.1111/jonm.13380

[nhs70352-bib-0031] Gottlieb, M. , T. M. Chan , F. Zaver , and R. Ellaway . 2022. “Confidence‐Competence Alignment and the Role of Self‐Confidence in Medical Education: A Conceptual Review.” Medical Education 56, no. 1: 37–47. 34176144 10.1111/medu.14592

[nhs70352-bib-0032] Greenland, S. , and K. O'Rourke . 2001. “On the Bias Produced by Quality Scores in Meta‐Analysis, and a Hierarchical View of Proposed Solutions.” Biostatistics 2, no. 4: 463–471. 12933636 10.1093/biostatistics/2.4.463

[nhs70352-bib-0033] Håkanson, C. , B. S. Cronfalk , E. Henriksen , A. Norberg , B. M. Ternestedt , and J. Sandberg . 2014. “First‐Line Nursing Home Managers in Sweden and Their Views on Leadership and Palliative Care.” Open Nursing Journal 8: 71–78. 25628769 10.2174/1874434601408010071 PMC4303953

[nhs70352-bib-0034] Hallberg, I. R. 2006. “Palliative Care as a Framework for Older People's Long‐Term Care.” International Journal of Palliative Nursing 12, no. 5: 224–229. 16835562 10.12968/ijpn.2006.12.5.21175

[nhs70352-bib-0035] Haycock‐Stuart, E. , and S. Kean . 2012. “Does Nursing Leadership Affect the Quality of Care in the Community Setting?” Journal of Nursing Management 20, no. 3: 372–381. 22519615 10.1111/j.1365-2834.2011.01309.x

[nhs70352-bib-0036] Henderson, S. E. M. , E. M. Brady , and N. Robertson . 2019. “Associations Between Social Jetlag and Mental Health in Young People: A Systematic Review.” Chronobiology International 36, no. 10: 1316–1333. 31387413 10.1080/07420528.2019.1636813

[nhs70352-bib-0037] Hökkä, M. , S. Martins Pereira , T. Pölkki , H. Kyngäs , and P. Hernández‐Marrero . 2020. “Nursing Competencies Across Different Levels of Palliative Care Provision: A Systematic Integrative Review With Thematic Synthesis.” Palliative Medicine 34, no. 7: 851–870. 32452294 10.1177/0269216320918798

[nhs70352-bib-0038] Hökkä, M. , H. L. Melender , J. T. Lehto , and P. Kaakinen . 2021. “Palliative Nursing Competencies Required for Different Levels of Palliative Care Provision: A Qualitative Analysis of Health Care Professionals' Perspectives.” Journal of Palliative Medicine 24, no. 10: 1516–1524. 33720785 10.1089/jpm.2020.0632 PMC8590151

[nhs70352-bib-0039] Hong, Q. N. P. P. , S. Fàbregues , G. Bartlett , et al. 2018. “Mixed Methods Appraisal Tool (MMAT), Version 2018. Registration of Copyright (#1148552),” Canada.

[nhs70352-bib-0040] Josefsson, K. , L. Sonde , and T. B. Wahlin . 2008. “Competence Development of Registered Nurses in Municipal Elderly Care in Sweden: A Questionnaire Survey.” International Journal of Nursing Studies 45, no. 3: 428–441. 17097089 10.1016/j.ijnurstu.2006.09.009

[nhs70352-bib-0041] Jüni, P. , A. Witschi , R. Bloch , and M. Egger . 1999. “The Hazards of Scoring the Quality of Clinical Trials for Meta‐Analysis.” JAMA 282, no. 11: 1054–1060. 10493204 10.1001/jama.282.11.1054

[nhs70352-bib-0042] Katz, J. , M. Sidell , and C. Komaromy . 2000. “Death in Homes: Bereavement Needs of Residents, Relatives and Staff.” International Journal of Palliative Nursing 6, no. 6: 274–279. 11249448 10.12968/ijpn.2000.6.6.9076

[nhs70352-bib-0043] Kröger, T. v A L. , and J. M. Puthenparambil . 2018. “Care Work in Transition.”

[nhs70352-bib-0044] Kvist, T. , K. Tähkä , M. Ruotsalainen , and T. Tervo‐Heikkinen . 2014. “The Impact of Nursing Leadership Training on Evidence‐Based Leadership and Practice.” Journal of Nursing Care 3, no. 4: 2167.

[nhs70352-bib-0045] Lavoie, M. , D. Blondeau , and I. Martineau . 2013. “The Integration of a Person‐Centered Approach in Palliative Care.” Palliative & Supportive Care 11, no. 6: 453–464. 23388553 10.1017/S1478951512000855

[nhs70352-bib-0046] Li, T. H. J. , and J. J. Deeks . 2019. “Chapter 5: Collecting Data.” In Cochrane Handbook for Systematic Reviews of Interventions Version 65, edited by J. P. T. T. J. Higgins , J. Chandler , M. Cumpston , T. Li , M. J. Page , and V. A. Welch . Cochrane.

[nhs70352-bib-0047] Liberati, A. , D. G. Altman , J. Tetzlaff , et al. 2009. “The PRISMA Statement for Reporting Systematic Reviews and Meta‐Analyses of Studies That Evaluate Healthcare Interventions: Explanation and Elaboration.” BMJ 339: b2700. 19622552 10.1136/bmj.b2700 PMC2714672

[nhs70352-bib-0048] Lockwood, C. , Z. Munn , and K. Porritt . 2015. “Qualitative Research Synthesis: Methodological Guidance for Systematic Reviewers Utilizing Meta‐Aggregation.” International Journal of Evidence‐Based Healthcare 13, no. 3: 179–187. 26262565 10.1097/XEB.0000000000000062

[nhs70352-bib-0049] Lugtenberg, M. , J. S. Burgers , and G. P. Westert . 2009. “Effects of Evidence‐Based Clinical Practice Guidelines on Quality of Care: A Systematic Review.” Quality & Safety in Health Care 18, no. 5: 385–392. 19812102 10.1136/qshc.2008.028043

[nhs70352-bib-0050] Lund, H. , C. B. Juhl , B. Nørgaard , et al. 2021. “Evidence‐Based Research Series‐Paper 2: Using an Evidence‐Based Research Approach Before a New Study Is Conducted to Ensure Value.” Journal of Clinical Epidemiology 129: 158–166. 32987159 10.1016/j.jclinepi.2020.07.019

[nhs70352-bib-0051] Ma, L.‐L. , Y.‐Y. Wang , Z.‐H. Yang , D. Huang , H. Weng , and X.‐T. Zeng . 2020. “Methodological Quality (Risk of Bias) Assessment Tools for Primary and Secondary Medical Studies: What Are They and Which Is Better?” Military Medical Research 7, no. 1: 7. 32111253 10.1186/s40779-020-00238-8 PMC7049186

[nhs70352-bib-0052] Maetens, A. , K. Beernaert , L. Deliens , R. Aubry , L. Radbruch , and J. Cohen . 2017. “Policy Measures to Support Palliative Care at Home: A Cross‐Country Case Comparison in Three European Countries.” Journal of Pain and Symptom Management 54, no. 4: 523–529. 28736105 10.1016/j.jpainsymman.2017.07.022

[nhs70352-bib-0053] Mahon, M. M. , and W. J. McAuley . 2010. “Oncology Nurses' Personal Understandings About Palliative Care.” Oncology Nursing Forum 37, no. 3: E141–E150. 20439199 10.1188/10.ONF.E141-E150

[nhs70352-bib-0054] Martins Pereira, S. , P. Hernández‐Marrero , H. R. Pasman , M. L. Capelas , P. Larkin , and A. L. Francke . 2021. “Nursing Education on Palliative Care Across Europe: Results and Recommendations From the EAPC Taskforce on Preparation for Practice in Palliative Care Nursing Across the EU Based on an Online‐Survey and Country Reports.” Palliative Medicine 35, no. 1: 130–141. 32912033 10.1177/0269216320956817

[nhs70352-bib-0055] McCormick, M. J. 2001. “Self‐Efficacy and Leadership Effectiveness: Applying Social Cognitive Theory to Leadership.” Journal of Leadership Studies 8, no. 1: 22–33.

[nhs70352-bib-0056] McGregor, S. 2008. “Neoliberalism and Health Care.” International Journal of Consumer Studies 25: 82–89.

[nhs70352-bib-0057] McSherry, R. , P. Pearce , K. Grimwood , and W. McSherry . 2012. “The Pivotal Role of Nurse Managers, Leaders and Educators in Enabling Excellence in Nursing Care.” Journal of Nursing Management 20, no. 1: 7–19. 22229897 10.1111/j.1365-2834.2011.01349.x

[nhs70352-bib-0058] Miltner, R. S. , A. Jukkala , M. A. Dawson , and P. A. Patrician . 2015. “Professional Development Needs of Nurse Managers.” Journal of Continuing Education in Nursing 46, no. 6: 252–258. 26057161 10.3928/00220124-20150518-01

[nhs70352-bib-0059] Mitchell, G. , J. McGreevy , D. H. Preshaw , J. Agnelli , and M. Diamond . 2016. “Care Home Managers' Knowledge of Palliative Care: A Northern Irish Study.” International Journal of Palliative Nursing 22, no. 5: 230–235. 27233010 10.12968/ijpn.2016.22.5.230

[nhs70352-bib-0060] Morrison, L. , B. Johnston , and M. Cooper . 2022. “Mixed Methods Systematic Review: Factors Influencing Research Activity Among Nurses in Clinical Practice.” Journal of Clinical Nursing 31, no. 17–18: 2450–2464. 34820932 10.1111/jocn.16133

[nhs70352-bib-0061] Munkeby, H. , G. Bratberg , and S. A. Devik . 2023. “Registered Nurses' Exposure to High Stress of Conscience in Long‐Term Care.” Nursing Ethics 30, no. 7–8: 1011–1024. 37163482 10.1177/09697330231167542 PMC10710004

[nhs70352-bib-0062] Nilsen, P. , B. Wallerstedt , L. Behm , and G. Ahlström . 2018. “Towards Evidence‐Based Palliative Care in Nursing Homes in Sweden: A Qualitative Study Informed by the Organizational Readiness to Change Theory.” Implementation Science 13, no. 1: 1. 29301543 10.1186/s13012-017-0699-0 PMC5753464

[nhs70352-bib-0063] Nilsson, K. , S. Lundgren , and C. Furåker . 2009. “Registered Nurses' Everyday Activities in Municipal Health Care: A Study of Diaries.” International Journal of Nursing Practice 15, no. 6: 543–552. 19958409 10.1111/j.1440-172X.2009.01777.x

[nhs70352-bib-0064] Nurmeksela, A. , S. Mikkonen , J. Kinnunen , and T. Kvist . 2021. “Relationships Between Nurse Managers' Work Activities, Nurses' Job Satisfaction, Patient Satisfaction, and Medication Errors at the Unit Level: A Correlational Study.” BMC Health Services Research 21, no. 1: 296. 33794875 10.1186/s12913-021-06288-5 PMC8017674

[nhs70352-bib-0065] Österlind, J. , and I. Henoch . 2021. “The 6S‐Model for Person‐Centred Palliative Care: A Theoretical Framework.” Nursing Philosophy 22, no. 2: e12334. 33089912 10.1111/nup.12334 PMC8243997

[nhs70352-bib-0066] Owens, K. , and S. Keller . 2018. “Exploring Workforce Confidence and Patient Experiences: A Quantitative Analysis.” Patient Experience Journal 5: 97–105.

[nhs70352-bib-0067] Page, M. J. , M. K. JE , P. M. Bossuyt , et al. 2021. “The PRISMA 2020 Statement: An Updated Guideline for Reporting Systematic Reviews.” BMJ 372: n71. 33782057 10.1136/bmj.n71 PMC8005924

[nhs70352-bib-0068] Payne, S. , A. Harding , T. Williams , J. Ling , and C. Ostgathe . 2022. “Revised Recommendations on Standards and Norms for Palliative Care in Europe From the European Association for Palliative Care (EAPC): A Delphi Study.” Palliative Medicine 36, no. 4: 680–697. 35114839 10.1177/02692163221074547 PMC9006395

[nhs70352-bib-0069] Pereira, D. G. , J. Fernandes , L. S. Ferreira , R. O. Rabelo , J. D. R. Pessalacia , and R. S. Souza . 2017. “Meanings of Palliative Care in the View of Nurses and Managers of Primary Health Care.” Journal of Nursing Ufpe Online 11: 1357–1364.

[nhs70352-bib-0070] Perez‐Gonzalez, S. , P. Marques‐Sanchez , A. Pinto‐Carral , A. Gonzalez‐Garcia , C. Liebana‐Presa , and C. Benavides . 2024. “Characteristics of Leadership Competency in Nurse Managers: A Scoping Review.” Journal of Nursing Management 2024: 5594154. 40224788 10.1155/2024/5594154 PMC11921696

[nhs70352-bib-0071] Phillips, J. L. , and M. R. Agar . 2016. “Exemplary Nursing Leadership Is Central to Improving Care of the Dying.” Journal of Nursing Management 24, no. 1: 1–3. 26781423 10.1111/jonm.12353

[nhs70352-bib-0072] Popay, J., H. M. Roberts , A. J. Sowden , et al. editors. 2006. “Guidance on the Conduct of Narrative Synthesis in Systematic Reviews. A Product From the ESRC Methods Programme,” Version 12006.

[nhs70352-bib-0073] Pope, C. , N. Mays , and J. Popay . 2007. Synthesising Qualitative and Quantitative Health Evidence: A Guide to Methods. Open University Press.

[nhs70352-bib-0074] Radbruch, L. , and P. Sa . 2010. “White Paper on Standards and Norms for Hospice and Palliative Care in Europe: Part 1.” European Journal of Palliative Care 17: 22–33.

[nhs70352-bib-0075] Randall, F. , and R. S. Downie . 2006. The Philosophy of Palliative Care: Critique and Reconstruction. Oxford University Press.

[nhs70352-bib-0076] Robinson, J. , R. Frey , G. Gibbs , M. Hayden , and M. Gott . 2023. “The Contribution of Generalist Community Nursing to Palliative Care: A Retrospective Case Note Review.” International Journal of Palliative Nursing 29, no. 2: 75–82. 36822619 10.12968/ijpn.2023.29.2.75

[nhs70352-bib-0077] Rosenthal, D. 2019. “Consciousness and Confidence.” Neuropsychologia 128: 255–265. 29355646 10.1016/j.neuropsychologia.2018.01.018

[nhs70352-bib-0078] Rostad, H. M. , L. V. Burrell , M. S. Skinner , R. Hellesø , and M. K. R. Sogstad . 2023. “Quality of Municipal Long‐Term Care in Different Models of Care: A Cross‐Sectional Study From Norway.” Health Services Insights 16: 11786329231185537. 37475731 10.1177/11786329231185537 PMC10354822

[nhs70352-bib-0079] Rovito, M. J. , A. Bruzzone , E. Lee , et al. 2021. “Assessing Health‐Related Quality of Life Among Survivors of Testicular Cancer: A Systematic Review.” American Journal of Men's Health 15, no. 1: 1557988320982184. 10.1177/1557988320982184 PMC7812415 33451261

[nhs70352-bib-0080] Sanderson, S. , I. D. Tatt , and J. P. Higgins . 2007. “Tools for Assessing Quality and Susceptibility to Bias in Observational Studies in Epidemiology: A Systematic Review and Annotated Bibliography.” International Journal of Epidemiology 36, no. 3: 666–676. 17470488 10.1093/ije/dym018

[nhs70352-bib-0081] Sandström, B. , G. Borglin , R. Nilsson , and A. Willman . 2011. “Promoting the Implementation of Evidence‐Based Practice: A Literature Review Focusing on the Role of Nursing Leadership.” Worldviews on Evidence‐Based Nursing 8, no. 4: 212–223. 21401858 10.1111/j.1741-6787.2011.00216.x

[nhs70352-bib-0082] Sawatzky, R. , P. Porterfield , J. Lee , et al. 2016. “Conceptual Foundations of a Palliative Approach: A Knowledge Synthesis.” BMC Palliative Care 15: 5. 26772180 10.1186/s12904-016-0076-9 PMC4715271

[nhs70352-bib-0083] Sawatzky, R. , P. Porterfield , D. Roberts , et al. 2017. “Embedding a Palliative Approach in Nursing Care Delivery: An Integrated Knowledge Synthesis.” ANS. Advances in Nursing Science 40, no. 3: 261–277. 27930401 10.1097/ANS.0000000000000163 PMC5555976

[nhs70352-bib-0084] Scott, S. D. , T. Rotter , R. Flynn , et al. 2019. “Systematic Review of the Use of Process Evaluations in Knowledge Translation Research.” Systematic Reviews 8, no. 1: 266. 31699136 10.1186/s13643-019-1161-y PMC6836407

[nhs70352-bib-0085] Shaw, R. L. , A. Booth , A. J. Sutton , et al. 2004. “Finding Qualitative Research: An Evaluation of Search Strategies.” BMC Medical Research Methodology 4: 5. 15070427 10.1186/1471-2288-4-5 PMC385230

[nhs70352-bib-0086] Silva, L. B. , M. H. O. Sousa , and L. Íñiguez‐Rueda . 2022. “Managers' Views on Professional Competencies for Primary Health Care.” SAGE Open 12, no. 4: 21582440221138252.

[nhs70352-bib-0087] Silva, T. C. D. , E. A. Nietsche , and S. B. Cogo . 2021. “Palliative Care in Primary Health Care: An Integrative Literature Review.” Revista Brasileira de Enfermagem 75, no. 1: e20201335. 34614078 10.1590/0034-7167-2020-1335

[nhs70352-bib-0088] Sneltvedt, T. , and T. Bondas . 2016. “Proud to be a Nurse? Recently Graduated Nurses' Experiences in Municipal Health Care Settings.” Scandinavian Journal of Caring Sciences 30, no. 3: 557–564. 26459623 10.1111/scs.12278

[nhs70352-bib-0090] Solbakken, R. , T. Bondas , and A. Kasén . 2019. “Safeguarding the Patient in Municipal Healthcare‐A Hermeneutic Focus Group Study of Nordic Nursing Leadership.” Journal of Nursing Management 27, no. 6: 1242–1250. 31136017 10.1111/jonm.12806

[nhs70352-bib-0091] Søreide, H. , D. Kyrkjebø , and M. B. Råholm . 2019. “Challenges in Municipality Healthcare Services‐The Nurse Leaders' Perspective.” Nursing Open 6, no. 3: 889–896. 31367412 10.1002/nop2.270 PMC6650667

[nhs70352-bib-0092] Thomas, J. , and A. Harden . 2008. “Methods for the Thematic Synthesis of Qualitative Research in Systematic Reviews.” BMC Medical Research Methodology 8: 45. 18616818 10.1186/1471-2288-8-45 PMC2478656

[nhs70352-bib-0093] Tunnard, I. , K. E. Sleeman , A. Bradshaw , A. E. Bone , and C. J. Evans . 2024. “The Influence of Care Home Registration Type and Size on Senior Care Leader's Confidence to Provide Palliative and End‐Of‐Life Care: An Explanatory Sequential Mixed Methods Study.” BMC Palliative Care 23, no. 1: 213. 39174986 10.1186/s12904-024-01525-0 PMC11340158

[nhs70352-bib-0094] Van Dyk, J. , S. L. Siedlecki , and J. J. Fitzpatrick . 2016. “Frontline Nurse Managers' Confidence and Self‐Efficacy.” Journal of Nursing Management 24, no. 4: 533–539. 26762223 10.1111/jonm.12355

[nhs70352-bib-0095] Vesterinen, S. , M. Suhonen , A. Isola , L. Paasivaara , and H. Laukkala . 2013. “Nurse Managers' Perceptions Related to Their Leadership Styles, Knowledge, and Skills in These Areas‐A Viewpoint: Case of Health Centre Wards in Finland.” ISRN Nursing 2013: 951456. 23691356 10.1155/2013/951456 PMC3649531

[nhs70352-bib-0096] Wallerstedt, B. , E. Benzein , K. Schildmeijer , and A. Sandgren . 2019. “What Is Palliative Care? Perceptions of Healthcare Professionals.” Scandinavian Journal of Caring Sciences 33, no. 1: 77–84. 30101989 10.1111/scs.12603 PMC7432164

[nhs70352-bib-0097] Webb, T. W. , H. H. Kean , and M. S. Graziano . 2016. “Effects of Awareness on the Control of Attention.” Journal of Cognitive Neuroscience 28, no. 6: 842–851. 26836517 10.1162/jocn_a_00931

[nhs70352-bib-0098] Whittaker, A. L. , R. P. George , and L. O'Malley . 2022. “Prevalence of Cognitive Impairment Following Chemotherapy Treatment for Breast Cancer: A Systematic Review and Meta‐Analysis.” Scientific Reports 12, no. 1: 2135. 35136066 10.1038/s41598-022-05682-1 PMC8826852

[nhs70352-bib-0099] Wilkinson, J. E. , S. M. Nutley , and H. T. O. Davies . 2011. “An Exploration of the Roles of Nurse Managers in Evidence‐Based Practice Implementation.” Worldviews on Evidence‐Based Nursing 8, no. 4: 236–246. 21668735 10.1111/j.1741-6787.2011.00225.x

[nhs70352-bib-0100] Wong, J. N. , E. McAuley , and L. Trinh . 2018. “Physical Activity Programming and Counseling Preferences Among Cancer Survivors: A Systematic Review.” International Journal of Behavioral Nutrition and Physical Activity 15, no. 1: 48. 29879993 10.1186/s12966-018-0680-6 PMC5992647

[nhs70352-bib-0101] World Health Organisation . 2018. Integrating Palliative Care and Symptom Relief Into Primary Health Care: A WHO Guide for Planners, Implementers and Managers. World Health Organization.

[nhs70352-bib-0102] World Health Organisation . 2020. “Palliative Care: World Health Organisation.” https://www.who.int/news‐room/fact‐sheets/detail/palliative‐care.

[nhs70352-bib-0103] World Health Organisation . 2023. “Building Health Workforce Leadership Capacity.” https://www.who.int/europe/activities/building‐health‐workforce‐leadership‐capacity.

