# Supplementary material for: Nurse Managers' Awareness of and Competence in Palliative Care in Municipal Care Settings: A Systematic Mixed Studies Review
Source: Nurs Health Sci. 2026 May 12;28:e70352. doi: 10.1111/nhs.70352 (PMC13167248; doi:10.1111/nhs.70352)
Supplement: Supplementary file 2 — File S2: Search strategy. [file NHS-28-e70352-s001.docx]

**Supplementary file 2 Search strategy**

Last search date: 2024-10-24

**PubMed**

| **SPIDER model** | **Search terms** | | **Search outcomes** |
| --- | --- | --- | --- |
| **Sample** | #1 | (nurse*[Title/Abstract] OR nursing[Title/Abstract] OR care[Title/Abstract] OR caring*[Title/Abstract]) AND (manager*[Title/Abstract] OR leader*[Title/Abstract] OR chief*[Title/Abstract] OR executive*[Title/Abstract] OR head*[Title/Abstract] OR director*[Title/Abstract] OR administrator*[Title/Abstract] OR principal*[Title/Abstract] OR boss*[Title/Abstract]) OR "Nurse Administrators"[Mesh] | 171,268 |
| **Phenomenon of Interest** | #2 | "Community Health Services"[Mesh] OR "Residential Facilities"[Mesh] OR "Local Government"[Mesh] OR "Community Health Nursing"[Mesh] OR municipal*[Title/Abstract] OR communit*[Title/Abstract] OR local governmen*[Title/Abstract] OR local authorit*[Title/Abstract] OR local health authorit*[Title/Abstract] OR local public[title/abstract] OR home*[Title/Abstract] OR residential[Title/Abstract] OR accommodation*[Title/Abstract] OR District*[Title/Abstract] OR County[Title/Abstract] OR Counties[Title/Abstract] OR city[Title/Abstract] OR cities[Title/Abstract] | 2,096,564 |
| **Evaluation** | #3 | "Palliative Care"[Mesh] OR "Hospice and Palliative Care Nursing"[Mesh] OR "Terminal Care"[Mesh] OR palliative[Title/Abstract] OR Hospice[Title/Abstract] OR end-of-life[Title/Abstract] OR end of life[Title/Abstract] OR terminal[Title/Abstract] | 622,224 |
|  | #4 | #1 AND #2 AND #3 | 1,795 |

**CINAHL complete**

| **SPIDER model** | **Search terms** | | **Search outcomes** |
| --- | --- | --- | --- |
| **Sample** | S1 | (TI manager* OR AB manager* OR TI leader* OR AB leader* OR TI chief* OR AB chief* OR TI head* OR AB head* OR TI executive* OR AB executive* OR TI director* OR AB director* OR TI administrator* OR AB administrator* OR TI principal* OR AB principal* OR TI boss* OR AB boss*) AND (TI nurse* OR AB nurse* OR TI nursing OR AB nursing OR TI care OR AB care OR TI caring OR AB caring) OR (MH "Nursing Leaders+" OR MH "Clinical Nurse Leaders" OR MH "Nurse Executives+" OR MH "Case Managers+") | 129,876 |
| **Phenomenon of Interest** | S2 | MH "Community Health Services" OR MH "Local Government" OR MH "Residential Care" OR MH "Home Nursing" OR MH "Home Health Nursing" OR TI municipal*” OR AB municipal* OR TI communit* OR AB communit* OR TI "local governmen*" OR AB "local governmen*" OR TI "local authorit*" OR AB "local authorit*" OR TI "local health authorit*" OR AB "local health authorit*” OR TI "local public" OR AB "local public” OR TI home* OR AB home* OR TI residential OR AB residential OR TI accommodation* OR AB accommodation* OR TI District* OR AB District* OR TI County OR AB County OR TI Counties OR AB Counties OR TI city OR AB city OR TI cities OR AB cities | 380,083 |
| **Evaluation** | S3 | MH "Palliative Care" OR MH "Hospice Nursing" OR MH "Hospice Care" OR MH "Palliative Care Nursing" OR MH "Terminal Care" OR TI palliative OR AB palliative OR TI Hospice OR AB Hospice OR TI "end-of-life" OR AB "end-of-life" OR TI "end of life" OR AB "end of life" OR TI terminal OR AB terminal | 109,683 |
|  | S4 | S1 AND S2 AND S3 | 917 |
|  | Filter | Peer reviewed | 820 |

**PsycINFO**

| **SPIDER model** | **Search terms** | | **Search outcomes** |
| --- | --- | --- | --- |
| **Sample** | #1 | (TI manager* OR AB manager* OR TI leader* OR AB leader* OR TI chief* OR AB chief* OR TI head* OR AB head* OR TI executive* OR AB executive* OR TI director* OR AB director* OR TI administrator* OR AB administrator* OR TI principal* OR AB principal* OR TI boss* OR AB boss*) AND (TI nurse* OR AB nurse* OR TI nursing OR AB nursing OR TI care OR AB care OR TI caring OR AB caring) | 46,611 |
| **Phenomenon of Interest** | #2 | DE "Community Health" OR DE "Community Welfare Services" OR DE "Community Services" OR DE "Nursing Homes" OR DE "Residential Care Institutions" OR TI municipal* OR AB municipal* OR TI communit* OR AB communit* OR TI "local governmen*" OR AB "local governmen*" OR TI "local authorit*" OR AB "local authorit*" OR TI "local health authorit*" OR AB "local health authorit*” OR TI "local public" OR AB "local public” OR TI home* OR AB home* OR TI residential OR AB residential OR TI accommodation* OR AB accommodation* OR TI District* OR AB District* OR TI County OR AB County OR TI Counties OR AB Counties OR TI city OR AB city OR TI cities OR AB cities | 670,602 |
| **Evaluation** | #3 | DE "Palliative Care" OR DE "Hospice" OR TI palliative OR AB palliative OR TI Hospice OR AB Hospice OR TI "end-of-life" OR AB "end-of-life" OR TI "end of life" OR AB "end of life" OR TI terminal OR AB terminal | 47,114 |
|  | #4 | #1 AND #2 AND #3 | 575 |
|  | Filter | Peer reviewed | 498 |

**Scopus**

| **SPIDER model** | **Search terms** | | **Search outcomes** |
| --- | --- | --- | --- |
| **Sample** | 1 | (TITLE-ABS-KEY ( manager* ) OR TITLE-ABS-KEY ( leader* ) OR TITLE-ABS-KEY ( chief* ) OR TITLE-ABS-KEY ( executive* ) OR TITLE-ABS-KEY ( head* ) OR TITLE-ABS-KEY ( director* ) OR TITLE-ABS-KEY ( administrator* ) OR TITLE-ABS-KEY ( principal* ) OR TITLE-ABS-KEY ( boss* )) AND (TITLE-ABS-KEY ( nursing ) OR TITLE-ABS-KEY ( nurse* ) OR TITLE-ABS-KEY ( care ) OR TITLE-ABS-KEY ( caring )) | 393,623 |
| **Phenomenon of Interest** | 2 | TITLE-ABS-KEY( municipal* ) OR TITLE-ABS-KEY( communit* ) OR TITLE-ABS-KEY( "local governmen*" ) OR TITLE-ABS-KEY( "local authorit*" ) OR TITLE-ABS-KEY( "local health authorit*" ) OR TITLE-ABS-KEY( "local public" ) OR TITLE-ABS-KEY ( home* ) OR TITLE-ABS-KEY ( residential ) OR TITLE-ABS-KEY ( accommodation* ) OR TITLE-ABS-KEY ( district* ) OR TITLE-ABS-KEY ( county ) OR TITLE-ABS-KEY ( counties ) OR TITLE-ABS-KEY ( city ) OR TITLE-ABS-KEY ( cities ) | 5,362,198 |
| **Evaluation** | 3 | TITLE-ABS-KEY ( terminal ) OR TITLE-ABS-KEY ( Hospice ) OR TITLE-ABS-KEY ( Palliative ) OR TITLE-ABS-KEY ( “end-of-life” ) OR TITLE-ABS-KEY ( “end of life” ) | 1,152,087 |
|  | 4 | 1 AND 2 AND 3 | 2,712 |

**ABI/INFORM Global**

| **SPIDER model** | **Search terms** | | **Search outcomes** |
| --- | --- | --- | --- |
| **Sample** | #1 | (title(nurse*) OR abstract(nurse*) OR title(nursing) OR abstract(nursing) OR title(care) OR abstract(care) OR title(caring) OR abstract(caring)) AND (title(manager*) OR abstract(manager*) OR title(leader*) OR abstract(leader*) OR title(chief*) OR abstract(chief*) OR title(head*) OR abstract(head*) OR title(executive*) OR abstract(executive*) OR title(director*) OR abstract(director*) OR title(administrator* ) OR abstract(administrator* ) OR title(principal*) OR abstract(principal*) OR title(boss*) OR abstract(boss*)) | 58,491 |
| **Phenomenon and Interest** | #2 | MAINSUBJECT.EXACT("Community health care") OR MAINSUBJECT.EXACT("Community nursing") OR MAINSUBJECT.EXACT("Nursing homes") OR MAINSUBJECT.EXACT("Municipal government") OR MAINSUBJECT.EXACT("Local government") OR MAINSUBJECT.EXACT("Districts") OR MAINSUBJECT.EXACT("City county") OR MAINSUBJECT.EXACT("Municipalities") OR title (municipal* ) OR abstract (municipal* ) OR title (communit* ) OR abstract (communit* ) OR title("local governmen*") OR abstract("local governmen*") OR title("local authorit*") OR abstract("local authorit*") OR title("local health authorit*") OR abstract("local health authorit*") OR title("local public") OR abstract("local public") OR title (home* ) OR abstract (home* ) OR title (residential ) OR abstract (residential ) OR title (accommodation* ) OR abstract (accommodation* ) OR title(district* ) OR abstract(district* ) OR title(county ) OR abstract(county ) OR title(counties ) OR abstract(counties ) OR title(city ) OR abstract(city ) OR title(cities ) OR abstract(cities ) | 2,463,248 |
| **Evaluation** | #3 | MAINSUBJECT.EXACT("Palliative care") OR MAINSUBJECT.EXACT("Hospice care") OR title (palliative) OR abstract (palliative) OR abstract (Hospice) OR title (Hospice) OR title (terminal) OR abstract (terminal) OR title ("end-of-life") OR abstract ("end-of-life") OR title ("end of life") OR abstract ("end of life") | 221,819 |
|  | #4 | #1 AND #2 AND #3 | 857 |
|  | Filter | Peer reviewed | 464 |

Updated search date: 2026-01-09

**PubMed**

| **SPIDER model** | **Search terms** | | **Search outcomes** |
| --- | --- | --- | --- |
| **Sample** | #1 | (nurse*[Title/Abstract] OR nursing[Title/Abstract] OR care[Title/Abstract] OR caring*[Title/Abstract]) AND (manager*[Title/Abstract] OR leader*[Title/Abstract] OR chief*[Title/Abstract] OR executive*[Title/Abstract] OR head*[Title/Abstract] OR director*[Title/Abstract] OR administrator*[Title/Abstract] OR principal*[Title/Abstract] OR boss*[Title/Abstract]) OR "Nurse Administrators"[Mesh] | 187,102 |
| **Phenomenon of Interest** | #2 | "Community Health Services"[Mesh] OR "Residential Facilities"[Mesh] OR "Local Government"[Mesh] OR "Community Health Nursing"[Mesh] OR municipal*[Title/Abstract] OR communit*[Title/Abstract] OR local governmen*[Title/Abstract] OR local authorit*[Title/Abstract] OR local health authorit*[Title/Abstract] OR local public[title/abstract] OR home*[Title/Abstract] OR residential[Title/Abstract] OR accommodation*[Title/Abstract] OR District*[Title/Abstract] OR County[Title/Abstract] OR Counties[Title/Abstract] OR city[Title/Abstract] OR cities[Title/Abstract] | 2,269,915 |
| **Evaluation** | #3 | "Palliative Care"[Mesh] OR "Hospice and Palliative Care Nursing"[Mesh] OR "Terminal Care"[Mesh] OR palliative[Title/Abstract] OR Hospice[Title/Abstract] OR end-of-life[Title/Abstract] OR end of life[Title/Abstract] OR terminal[Title/Abstract] | 647,141 |
|  | #4 | #1 AND #2 AND #3 | 1,952 |

**CINAHL complete**

| **SPIDER model** | **Search terms** | | **Search outcomes** |
| --- | --- | --- | --- |
| **Sample** | S1 | (TI manager* OR AB manager* OR TI leader* OR AB leader* OR TI chief* OR AB chief* OR TI head* OR AB head* OR TI executive* OR AB executive* OR TI director* OR AB director* OR TI administrator* OR AB administrator* OR TI principal* OR AB principal* OR TI boss* OR AB boss*) AND (TI nurse* OR AB nurse* OR TI nursing OR AB nursing OR TI care OR AB care OR TI caring OR AB caring) OR (MH "Nursing Leaders+" OR MH "Clinical Nurse Leaders" OR MH "Nurse Executives+" OR MH "Case Managers+") | 137,749 |
| **Phenomenon of Interest** | S2 | MH "Community Health Services" OR MH "Local Government" OR MH "Residential Care" OR MH "Home Nursing" OR MH "Home Health Nursing" OR TI municipal*” OR AB municipal* OR TI communit* OR AB communit* OR TI "local governmen*" OR AB "local governmen*" OR TI "local authorit*" OR AB "local authorit*" OR TI "local health authorit*" OR AB "local health authorit*” OR TI "local public" OR AB "local public” OR TI home* OR AB home* OR TI residential OR AB residential OR TI accommodation* OR AB accommodation* OR TI District* OR AB District* OR TI County OR AB County OR TI Counties OR AB Counties OR TI city OR AB city OR TI cities OR AB cities | 404,256 |
| **Evaluation** | S3 | MH "Palliative Care" OR MH "Hospice Nursing" OR MH "Hospice Care" OR MH "Palliative Care Nursing" OR MH "Terminal Care" OR TI palliative OR AB palliative OR TI Hospice OR AB Hospice OR TI "end-of-life" OR AB "end-of-life" OR TI "end of life" OR AB "end of life" OR TI terminal OR AB terminal | 115,700 |
|  | S4 | S1 AND S2 AND S3 | 977 |
|  | Filter | Peer reviewed | 878 |

**PsycINFO**

| **SPIDER model** | **Search terms** | | **Search outcomes** |
| --- | --- | --- | --- |
| **Sample** | #1 | (TI manager* OR AB manager* OR TI leader* OR AB leader* OR TI chief* OR AB chief* OR TI head* OR AB head* OR TI executive* OR AB executive* OR TI director* OR AB director* OR TI administrator* OR AB administrator* OR TI principal* OR AB principal* OR TI boss* OR AB boss*) AND (TI nurse* OR AB nurse* OR TI nursing OR AB nursing OR TI care OR AB care OR TI caring OR AB caring) | 49,632 |
| **Phenomenon of Interest** | #2 | DE "Community Health" OR DE "Community Welfare Services" OR DE "Community Services" OR DE "Nursing Homes" OR DE "Residential Care Institutions" OR TI municipal* OR AB municipal* OR TI communit* OR AB communit* OR TI "local governmen*" OR AB "local governmen*" OR TI "local authorit*" OR AB "local authorit*" OR TI "local health authorit*" OR AB "local health authorit*” OR TI "local public" OR AB "local public” OR TI home* OR AB home* OR TI residential OR AB residential OR TI accommodation* OR AB accommodation* OR TI District* OR AB District* OR TI County OR AB County OR TI Counties OR AB Counties OR TI city OR AB city OR TI cities OR AB cities | 706,430 |
| **Evaluation** | #3 | DE "Palliative Care" OR DE "Hospice" OR TI palliative OR AB palliative OR TI Hospice OR AB Hospice OR TI "end-of-life" OR AB "end-of-life" OR TI "end of life" OR AB "end of life" OR TI terminal OR AB terminal | 48,813 |
|  | #4 | #1 AND #2 AND #3 | 609 |
|  | Filter | Peer reviewed | 524 |

**Scopus**

| **SPIDER model** | **Search terms** | | **Search outcomes** |
| --- | --- | --- | --- |
| **Sample** | 1 | (TITLE-ABS-KEY ( manager* ) OR TITLE-ABS-KEY ( leader* ) OR TITLE-ABS-KEY ( chief* ) OR TITLE-ABS-KEY ( executive* ) OR TITLE-ABS-KEY ( head* ) OR TITLE-ABS-KEY ( director* ) OR TITLE-ABS-KEY ( administrator* ) OR TITLE-ABS-KEY ( principal* ) OR TITLE-ABS-KEY ( boss* )) AND (TITLE-ABS-KEY ( nursing ) OR TITLE-ABS-KEY ( nurse* ) OR TITLE-ABS-KEY ( care ) OR TITLE-ABS-KEY ( caring )) | 429,256 |
| **Phenomenon of Interest** | 2 | TITLE-ABS-KEY( municipal* ) OR TITLE-ABS-KEY( communit* ) OR TITLE-ABS-KEY( "local governmen*" ) OR TITLE-ABS-KEY( "local authorit*" ) OR TITLE-ABS-KEY( "local health authorit*" ) OR TITLE-ABS-KEY( "local public" ) OR TITLE-ABS-KEY ( home* ) OR TITLE-ABS-KEY ( residential ) OR TITLE-ABS-KEY ( accommodation* ) OR TITLE-ABS-KEY ( district* ) OR TITLE-ABS-KEY ( county ) OR TITLE-ABS-KEY ( counties ) OR TITLE-ABS-KEY ( city ) OR TITLE-ABS-KEY ( cities ) | 5,927,103 |
| **Evaluation** | 3 | TITLE-ABS-KEY ( terminal ) OR TITLE-ABS-KEY ( Hospice ) OR TITLE-ABS-KEY ( Palliative ) OR TITLE-ABS-KEY ( “end-of-life” ) OR TITLE-ABS-KEY ( “end of life” ) | 1,213,261 |
|  | 4 | 1 AND 2 AND 3 | 2,972 |

**ABI/INFORM Global**

| **SPIDER model** | **Search terms** | | **Search outcomes** |
| --- | --- | --- | --- |
| **Sample** | #1 | (title(nurse*) OR abstract(nurse*) OR title(nursing) OR abstract(nursing) OR title(care) OR abstract(care) OR title(caring) OR abstract(caring)) AND (title(manager*) OR abstract(manager*) OR title(leader*) OR abstract(leader*) OR title(chief*) OR abstract(chief*) OR title(head*) OR abstract(head*) OR title(executive*) OR abstract(executive*) OR title(director*) OR abstract(director*) OR title(administrator* ) OR abstract(administrator* ) OR title(principal*) OR abstract(principal*) OR title(boss*) OR abstract(boss*)) | 61,045 |
| **Phenomenon and Interest** | #2 | MAINSUBJECT.EXACT("Community health care") OR MAINSUBJECT.EXACT("Community nursing") OR MAINSUBJECT.EXACT("Nursing homes") OR MAINSUBJECT.EXACT("Municipal government") OR MAINSUBJECT.EXACT("Local government") OR MAINSUBJECT.EXACT("Districts") OR MAINSUBJECT.EXACT("City county") OR MAINSUBJECT.EXACT("Municipalities") OR title (municipal* ) OR abstract (municipal* ) OR title (communit* ) OR abstract (communit* ) OR title("local governmen*") OR abstract("local governmen*") OR title("local authorit*") OR abstract("local authorit*") OR title("local health authorit*") OR abstract("local health authorit*") OR title("local public") OR abstract("local public") OR title (home* ) OR abstract (home* ) OR title (residential ) OR abstract (residential ) OR title (accommodation* ) OR abstract (accommodation* ) OR title(district* ) OR abstract(district* ) OR title(county ) OR abstract(county ) OR title(counties ) OR abstract(counties ) OR title(city ) OR abstract(city ) OR title(cities ) OR abstract(cities ) | 2,603,660 |
| **Evaluation** | #3 | MAINSUBJECT.EXACT("Palliative care") OR MAINSUBJECT.EXACT("Hospice care") OR title (palliative) OR abstract (palliative) OR abstract (Hospice) OR title (Hospice) OR title (terminal) OR abstract (terminal) OR title ("end-of-life") OR abstract ("end-of-life") OR title ("end of life") OR abstract ("end of life") | 235,699 |
|  | #4 | #1 AND #2 AND #3 | 930 |
|  | Filter | Peer reviewed | 520 |
